# Supplementary material for: Hypoxia-induced conversion of sensory Schwann cells into repair cells is regulated by HDAC8
Source: Nat Commun. 2025 Jan 9;16:515. doi: 10.1038/s41467-025-55835-9 (PMC11711395; doi:10.1038/s41467-025-55835-9)
Supplement: Supplementary file 1 — Supplementary Information [file 41467_2025_55835_MOESM1_ESM.pdf]

Supplementary information

Hypoxia-Induced Conversion of Sensory Schwann Cells into Repair Cells is Regulated by HDAC8

Hertzog et al.

Supplementary Figures 1-19

Supplementary Data 1

Source Data folder

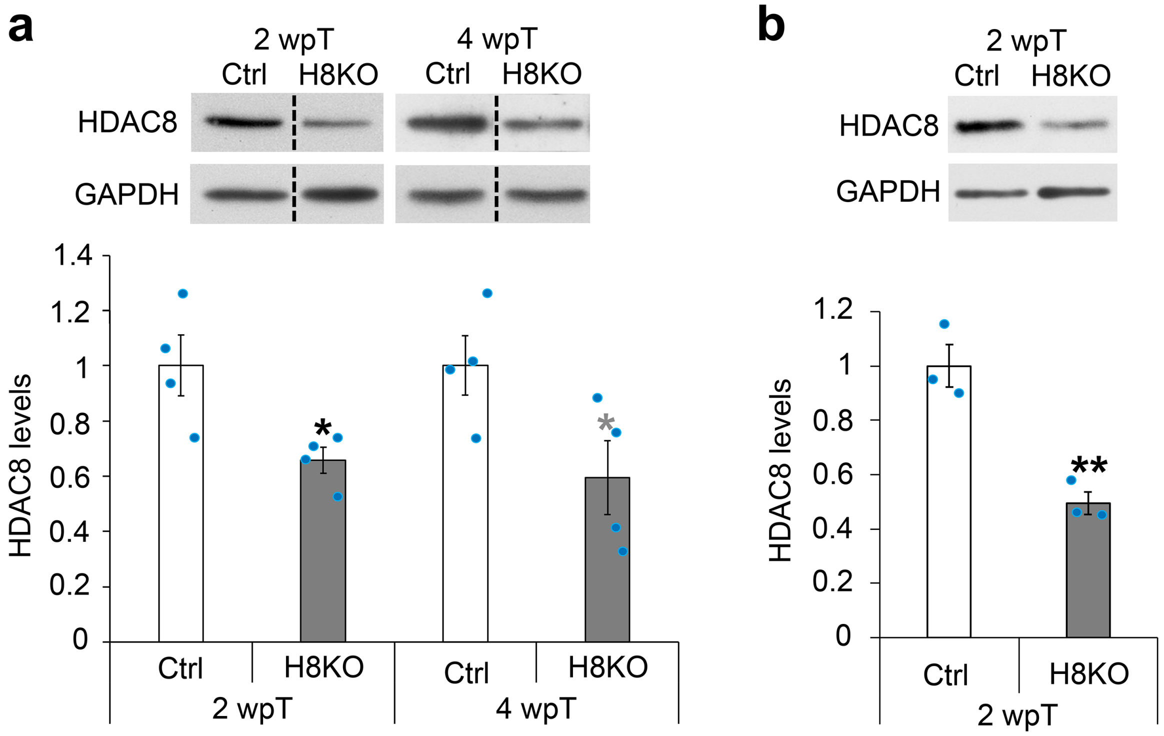

Supplementary Fig. 1. Onset of HDAC8 loss after tamoxifen injections. a, **b** HDAC8 Western blots on lysates of HDAC8 KO (H8KO) and control (Ctrl) sciatic nerves at 2 and 4 weeks post tamoxifen injections (wpT), and quantification normalized to GAPDH showing efficient HDAC8 recombination at both 2 and 4 wpT. Recombination efficiency is shown in both lines PLPCre-ERT2;*Hdac8* fl/fl (a) and in P0Cre-ERT2;*Hdac8* fl/fl (b) mice compared to their respective control littermates. Unpaired two-tailed (black asterisks) or one-tailed (grey asterisk) Student's t-tests, p values: *<0.05, **<0.01, values=mean, error bars=s.e.m., n=4 animals per group. In (a), dashed lines indicate that samples were run on the same gel but not on consecutive lanes.

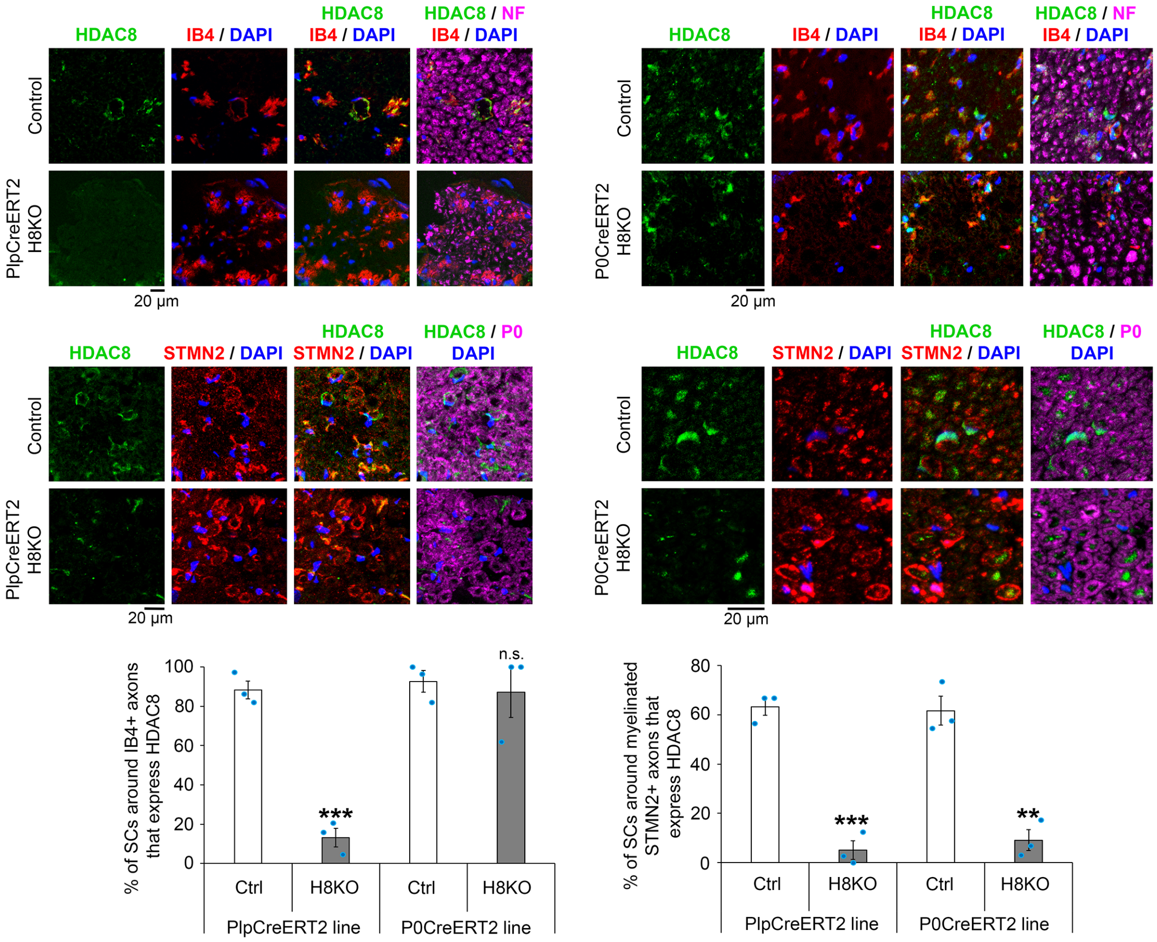

Supplementary Fig. 2. Recombination efficiency of HDAC8 in myelinating and non-myelinating sensory SCs in PlpCreERT2 HDAC8 KO and in P0CreERT2 HDAC8 KO nerves. Co-immunofluorescence of HDAC8 (green), Neurofilament (NF, magenta) or Myelin protein zero (P0, magenta), Stathmin-2 (red, STMN2, marker of sensory axons) or labeling of Isolectin B4 (red, IB4, marker of Remak axons), and DAPI labeling (blue, nuclei) in unlesioned sciatic nerves of PlpCreERT2 HDAC8 KO and P0CreERT2 HDAC8 KO and their respective control littermate mice. The quantifications show that around 90% of SCs surrounding IB4+ axons and around 60% of SCs surrounding myelinated Stathmin-2-positive axons express HDAC8. In addition, the quantifications also indicate high efficiency of HDAC8 recombination in SCs surrounding IB4+ and Stathmin-2+ axons in PlpCreERT2 HDAC8 KO sciatic nerves, high HDAC8 recombination efficiency in SCs surrounding Stathmin-2+ axons in P0CreERT2 HDAC8 KO sciatic nerves, but no recombination in SCs surrounding IB4+ axons. Unpaired two-tailed Student's t-tests, p values: $**<0.01$, $***<0.001$, n.s.=non-significant, values=mean, error bars=s.e.m., n=3 animals per group (all SCs surrounding either IB4+ or Stathmin-2+ axons in 1 to 3 sections per animal were counted).

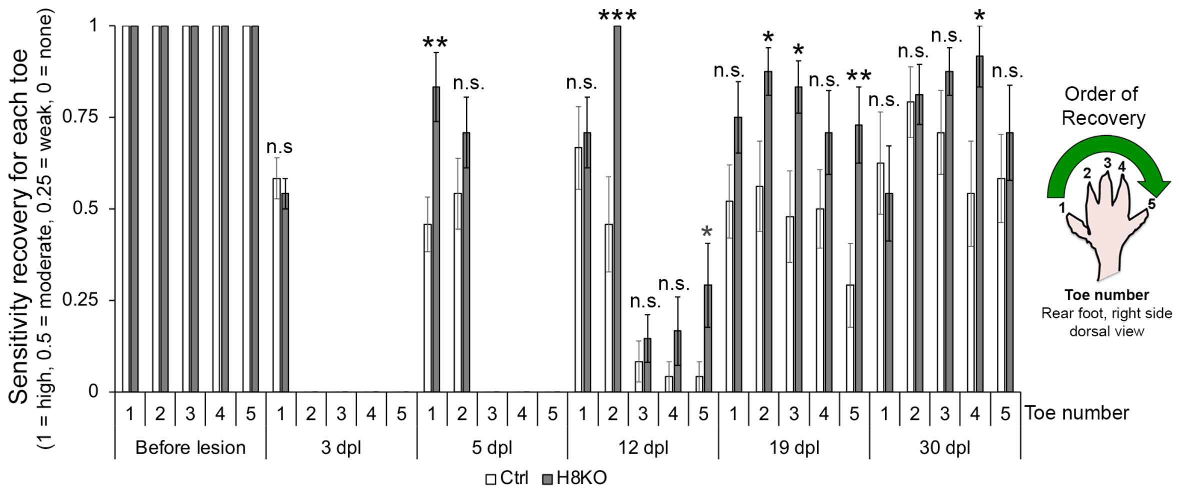

Individual data points

		Before lesion					3 dpl					5 dpl					12 dpl					19 dpl					30 dpl				
	Toe number	1	2	3	4	5	1	2	3	4	5	1	2	3	4	5	1	2	3	4	5	1	2	3	4	5	1	2	3	4	5
Sensitivity (1 = full, 0.5 = moderate, 0.25 = weak, 0 = none)	Ctrl-1	1	1	1	1	1	1	0	0	0	0	0.5	0.5	0	0	0	0.5	0.5	0.5	0.5	0.5	1	1	0.5	0.5	0	1	1	1	1	0.5
	Ctrl-2	1	1	1	1	1	1	0	0	0	0	0.5	1	0	0	0	1	0	0	0	0	0.5	0.5	0.5	0.5	0	1	1	1	0	0.5
	Ctrl-3	1	1	1	1	1	0.5	0	0	0	0	0.5	1	0	0	0	0	1	0	0	0	0.5	0.5	0	0	0	1	0.5	0.5	0	1
	Ctrl-4	1	1	1	1	1	0.5	0	0	0	0	0.5	1	0	0	0	1	1	0	0	0	1	1	0.5	1	0.5	1	1	1	1	1
	Ctrl-5	1	1	1	1	1	0.5	0	0	0	0	0.5	0.5	0	0	0	1	1	0	0	0	1	1	1	0.5	0.5	1	1	1	1	1
	Ctrl-6	1	1	1	1	1	0.5	0	0	0	0	0	0.5	0	0	0	0	0	0	0	0	0	0.5	0	0	1	0	0	0	0	0
	Ctrl-7	1	1	1	1	1	0.5	0	0	0	0	0.5	0.5	0	0	0	1	0.5	0	0	0	0.5	1	1	1	0	1	1	1	1	1
	Ctrl-8	1	1	1	1	1	0.5	0	0	0	0	0.5	0.5	0	0	0	0.5	0	0	0	0	0.25	0	0.25	0.5	0	0	0.5	0.5	0	0
	Ctrl-9	1	1	1	1	1	0.5	0	0	0	0	0	0	0	0	0	0.5	0	0	0	0	0.5	1	0	0	0.5	0.5	1	0.5	0.5	0.5
	Ctrl-10	1	1	1	1	1	0.5	0	0	0	0	1	0.5	0	0	0	0.5	0.5	0	0	0	0.5	0.25	0	0.5	0	1	1	1	1	1
	Ctrl-11	1	1	1	1	1	0.5	0	0	0	0	0.5	0	0	0	0	1	1	0.5	0	0	0.5	0	1	0.5	1	0	1	0	0	0
	Ctrl-12	1	1	1	1	1	0.5	0	0	0	0	0.5	0.5	0	0	0	1	0	0	0	0	0	0	1	1	0	0	0.5	1	1	0.5
	H8KO-1	1	1	1	1	1	1	0	0	0	0	0.5	0.5	0	0	0	1	1	0	1	1	0.5	1	1	1	1	1	1	1	1	1
	H8KO-2	1	1	1	1	1	0.5	0	0	0	0	0.5	0.5	0	0	0	1	1	0.5	0.5	1	1	0.5	1	1	0.25	1	1	1	1	0
	H8KO-3	1	1	1	1	1	0.5	0	0	0	0	1	1	0	0	0	0	1	0.5	0	0	0.5	0.5	0.5	0	0	0	0.5	1	1	1
	H8KO-4	1	1	1	1	1	0.5	0	0	0	0	1	1	0	0	0	0.5	1	0	0	0	1	1	1	0.5	0.5	0	0.5	1	1	0
	H8KO-5	1	1	1	1	1	0.5	0	0	0	0	1	1	0	0	0	1	1	0.25	0	0	1	0.5	0.5	1	1	0	0.5	0.5	0	0
	H8KO-6	1	1	1	1	1	0.5	0	0	0	0	1	0	0	0	0	1	1	0.5	0	0	1	1	1	1	0.5	1	1	1	1	1
	H8KO-7	1	1	1	1	1	0.5	0	0	0	0	1	1	0	0	0	1	1	0	0	0	0	1	0.5	0.5	1	0	0.25	0.5	1	0.5
	H8KO-8	1	1	1	1	1	0.5	0	0	0	0	0	0.5	0	0	0	0.5	1	0	0	0	1	1	1	1	0.5	0.5	1	1	1	1
	H8KO-9	1	1	1	1	1	0.5	0	0	0	0	1	0.5	0	0	0	0.5	1	0	0.5	0.5	0.5	1	1	1	1	1	1	0.5	1	1
	H8KO-10	1	1	1	1	1	0.5	0	0	0	0	1	1	0	0	0	0.5	1	0	0	0.5	1	1	1	0.5	1	0.5	1	1	1	1
	H8KO-11	1	1	1	1	1	0.5	0	0	0	0	1	0.5	0	0	0	1	1	0	0	0	1	1	1	0	1	1	1	1	1	1
	H8KO-12	1	1	1	1	1	0.5	0	0	0	0	1	1	0	0	0	0.5	1	0	0	0.5	0.5	1	0.5	1	1	0.5	1	1	1	1

Supplementary Fig. 3. Recovery of sensory function for each toe by Toe pinch test. Graph showing the sensitivity of each toe in HDAC8 KO and Ctrl mice before lesion or at 3, 5, 12, 19 and 30 dpl. Unpaired two-tailed (black asterisks) or one-tailed (grey asterisks and n.s.) Student's t-tests, p values: *<0.05, **<0.01, ***<0.001, n.s.=non-significant, values=mean, error bars=s.e.m., n=12 animals per group. Individual data points are shown in the table below the graph.

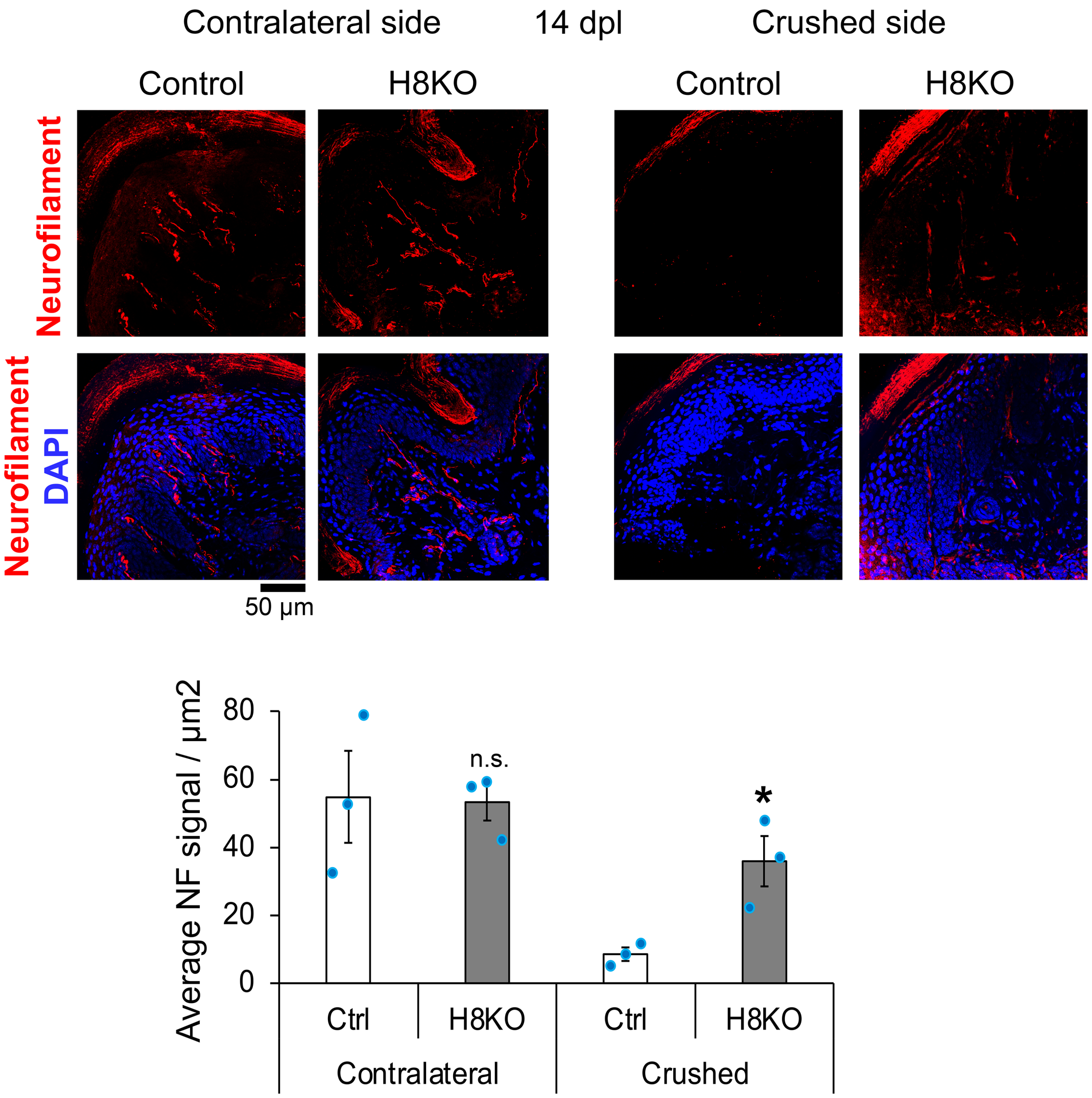

Supplementary Fig. 4. Re-innervation of hind paw skin after injury is promoted in the absence of HDAC8 in SCs. Immunofluorescence of Neurofilament (NF, red) and DAPI labeling (blue, nuclei) in hind paw skin on the crushed and contralateral sides of HDAC8 KO and control littermate mice at 14 dpl, and quantification of NF signal integrated density per μm^2. Unpaired two-tailed Student's t-tests, p values: *<0.05, n.s.=non-significant, values=mean, error bars=s.e.m., n=3 animals per group (signal integrated density of 2 to 8 sections per animal were analyzed using color range selection in Photoshop, and averaged). Of note, the stratum corneum (top layer) gives a strong autofluorescence signal that was excluded from the quantification.

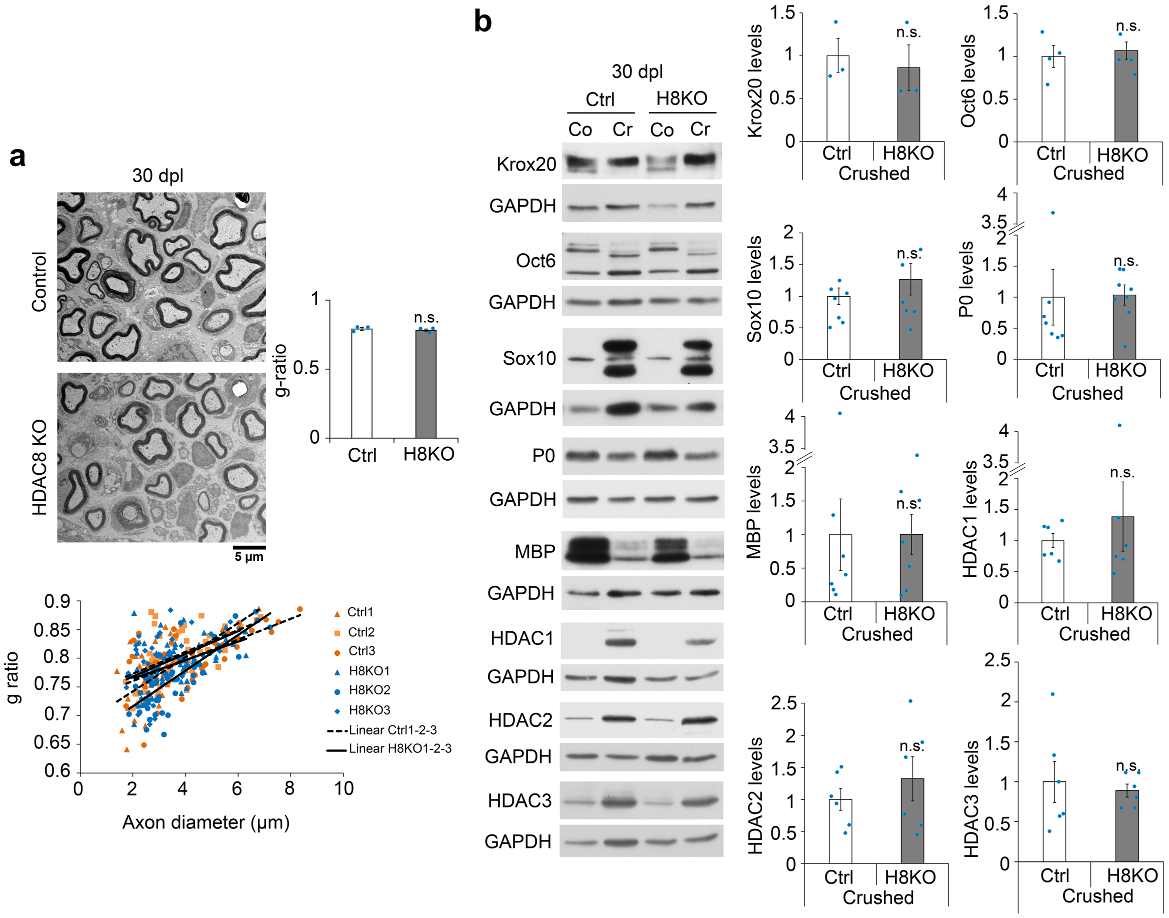

Supplementary Fig. 5. Ablation of HDAC8 does not affect remyelination after injury. a Electron micrographs of ultrathin cross sections from HDAC8 KO (H8KO) and Control (Ctrl) crushed sciatic nerves at 30 dpl, and quantification of g-ratio (=axon diameter/(axon+myelin) diameter) represented either as average of all counted fibers or plotted against the axon diameter, showing no significant difference of myelin thickness between H8KO and Ctrl nerves. Unpaired one-tailed Student's t-tests, n.s.=non significant, values=mean, error bars=s.e.m., n=3 animals per group, 50 myelinated axons randomly chosen from 3 different sections were quantified per animal. **b** Western blots of Krox20, Oct6, Sox10, P0, MBP, HDAC1, HDAC2 and HDAC3 on lysates of HDAC8 KO (H8KO) and control (Ctrl) sciatic nerves at 30 dpl, and quantification of protein levels in crushed nerves normalized to GAPDH and to contralateral nerves of the same mice, showing no significant difference between the two experimental groups. Unpaired one-tailed Student's t-tests, n.s.=non significant, values=mean, error bars=s.e.m., n=3 (Krox20), 4 (Oct6), 6 (Sox10, HDAC1, HDAC2, HDAC3), or 7 (P0 and MBP) animals per experimental group.

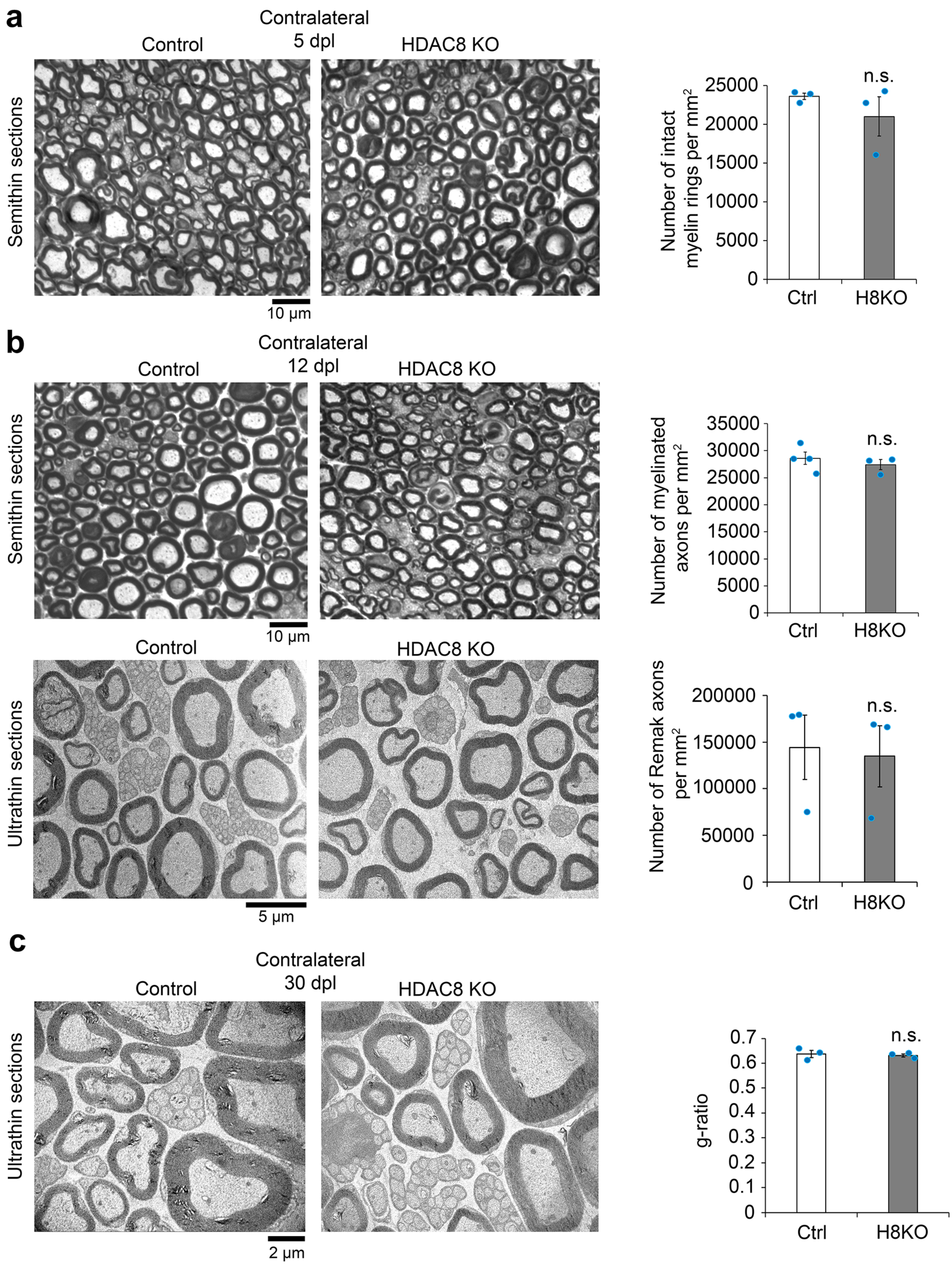

Supplementary Fig. 6. The contralateral nerve of HDAC8 KO mice is not affected. a Semithin cross-sections of HDAC8 KO (H8KO) and Control (Ctrl) sciatic nerves of adult mice at 5 dpl, and graphs showing the number of intact myelin rings per mm^2 (352 to 469 myelinated axons of 1 to 2 sections per animal were counted, no degenerated myelin ring was found). Unpaired one-tailed Student's t-tests, n.s.=non-significant, values=mean, error bars=s.e.m., n=3 animals per group. **b** Semithin (upper panel) and ultrathin (lower panel) cross-sections of H8KO and Ctrl sciatic nerves of adult mice at 12 dpl, and graphs showing the number of

myelinated and Remak axons per mm^2 (396 to 487 myelinated axons were counted per animal and no degenerated myelin ring was found). Unpaired one-tailed Student's t-tests, n.s.=non-significant, values=mean, error bars=s.e.m., n=3-4 animals per group. **c** Electron micrographs of ultrathin cross sections of H8KO and Ctrl crushed sciatic nerves at 30 dpl, and quantification of g-ratio (=axon diameter/(axon+myelin) diameter) showing no significant difference of myelin thickness between H8KO and Ctrl nerves. Unpaired one-tailed Student's t-tests, n.s.=non significant, values=mean, error bars=s.e.m., n=3 animals per group, 70 myelinated axons randomly chosen from 3 different sections were quantified per animal.

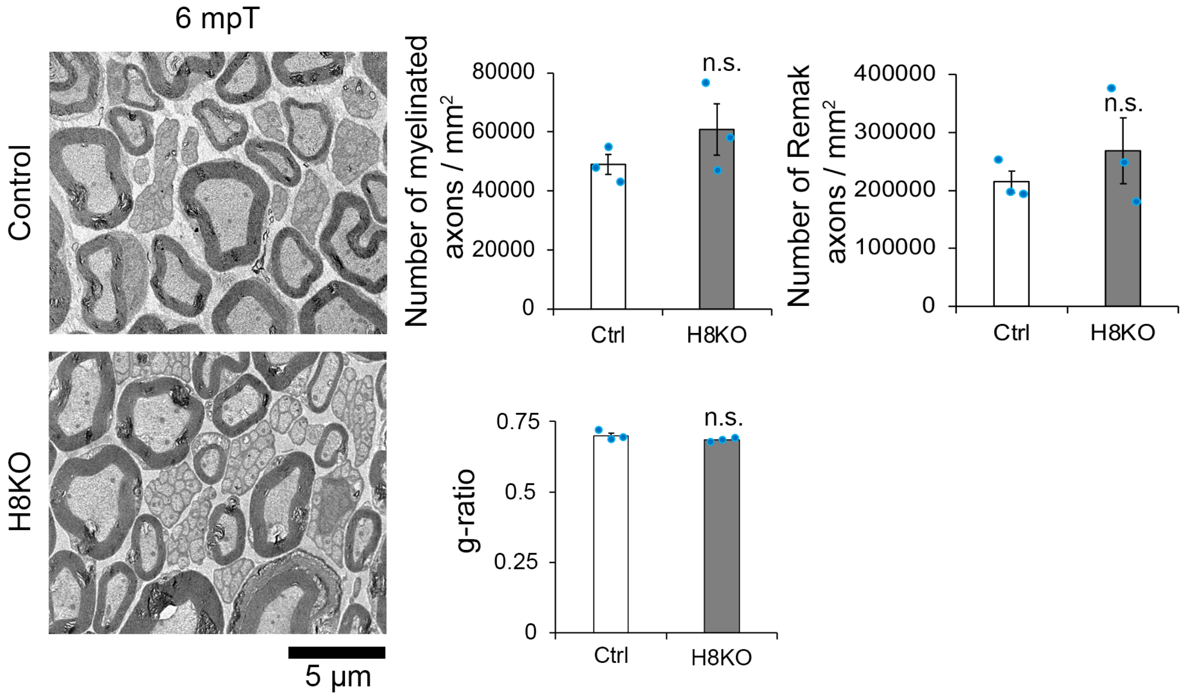

Supplementary Fig. 7. No maintenance defect in the absence of HDAC8 in SCs. Electron micrographs of ultrathin cross sections of H8KO (P0CreERT2;Hdac8 fl/fl) and Ctrl uninjured sciatic nerves at 6 months post tamoxifen injections (6 mpT), and quantification of myelinated and Remak axons per mm^2, and of g-ratio (=axon diameter/(axon+myelin) diameter) showing no significant difference between H8KO and Ctrl nerves. Unpaired one-tailed Student's t-tests, n.s.=non significant, values=mean, error bars=s.e.m., n=3 animals per group, 50 myelinated axons randomly chosen from 2 to 3 different sections were quantified per animal.

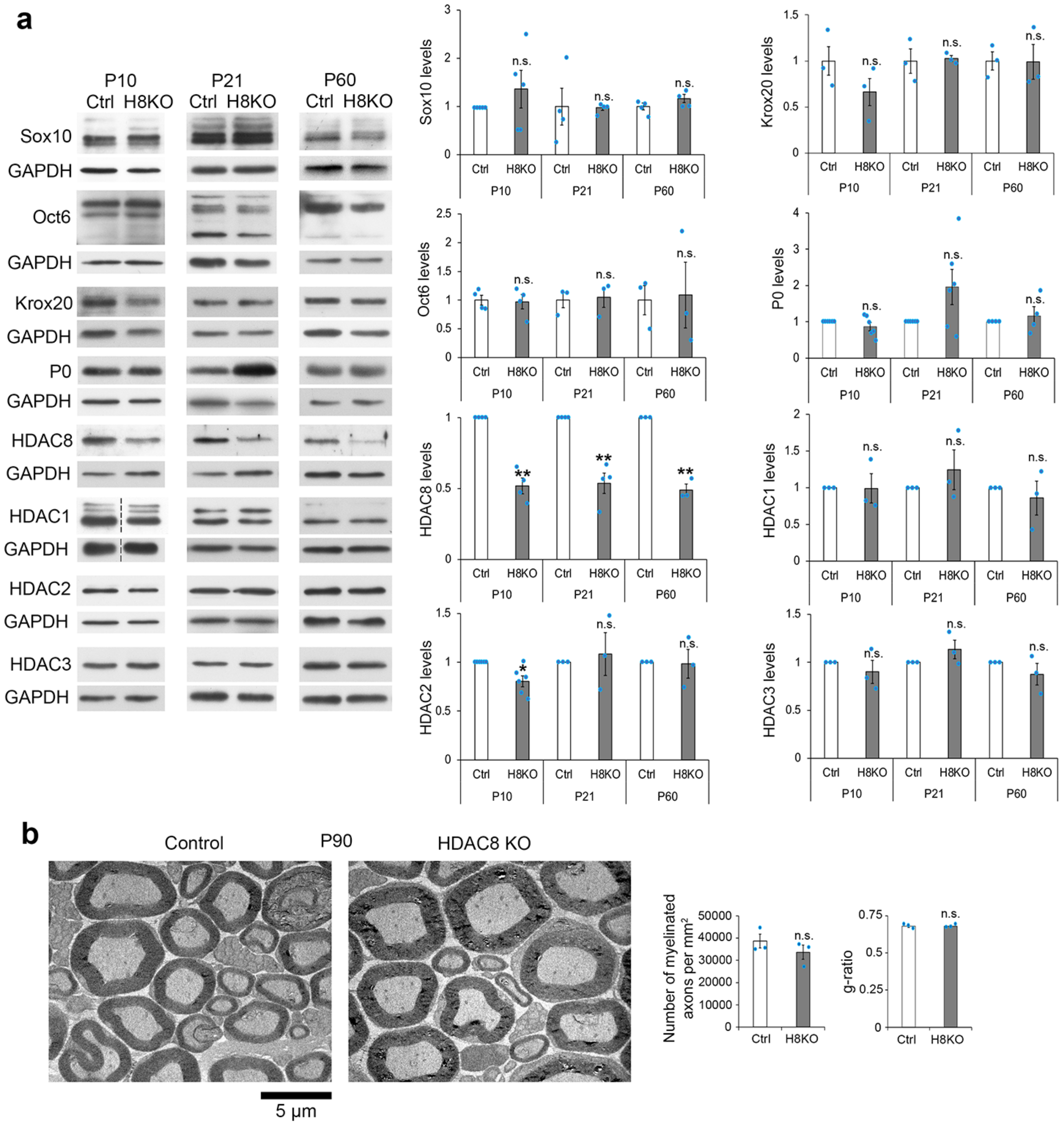

Supplementary Fig. 8. No developmental defect in the absence of HDAC8 in SCs. a Western blots of Sox10, Oct6, Krox20, P0, HDAC8, HDAC1, HDAC2 and HDAC3 on lysates of HDAC8 KO (H8KO, DhhCre;Hdac8 fl/fl) and control (Ctrl) sciatic nerves at postnatal day (P) 10, 21 and 60, and quantification of protein levels normalized to GAPDH. Unpaired (Sox10: P21, P60; Krox20, Oct6) or paired two-tailed (black asterisks) or one-tailed (n.s.) Student's t-tests, p values: *<0.05, **<0.001, n.s.=non significant, values=mean, error bars=s.e.m., n=3 to 6 animals per experimental group. **b** Electron micrographs of ultrathin cross sections of H8KO and Ctrl uninjured sciatic nerves at P90, and quantification of myelinated axons per mm^2, and of g-ratio (=axon diameter/(axon+myelin) diameter) showing no significant difference between H8KO and Ctrl nerves. Unpaired one-tailed Student's t-tests, n.s.=non significant, values=mean, error bars=s.e.m., n=3 animals per group, 50 myelinated axons randomly chosen from 1 to 2 different sections were quantified per animal.

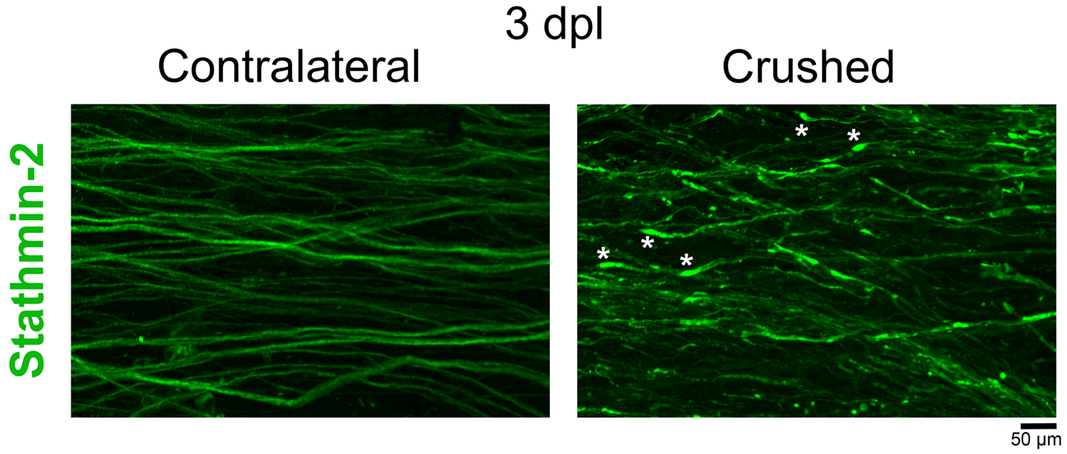

Supplementary Fig. 9. Stathmin-2 expression in injured and uninjured sciatic nerves. Immunofluorescence images of Stathmin-2 in crushed and contralateral sciatic nerves of the same adult mouse at 3 dpl (images taken at the same exposure). Longitudinal sections of 3 control mice were analyzed and representative images are shown. White asterisks indicate growth cones.

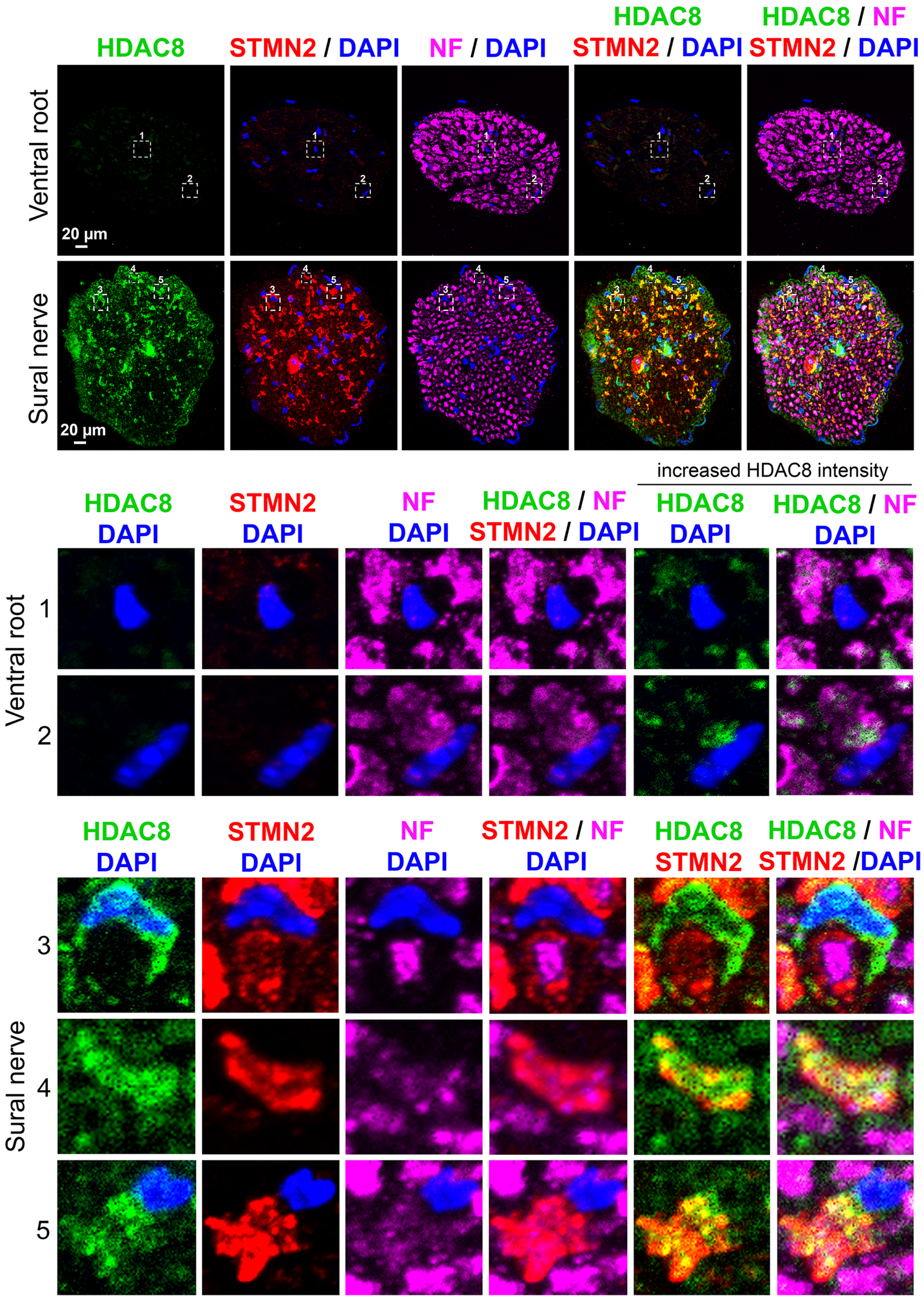

Supplementary Fig. 10. HDAC8 is abundantly expressed in SCs of sural nerves but is not detected in SCs of ventral roots. Co-immunofluorescence of HDAC8 (green), Neurofilament (NF, magenta), Stathmin-2 (STMN2, red) , and DAPI labeling (blue, nuclei) in ventral roots or sural nerves of wild type adult mice. Representative images of ventral roots and sural nerves from 3 different mice stained at the same time and imaged at the same exposure are shown in

the upper panel. The middle (ventral root) and lower (sural nerve) panels show magnifications delineated by the white dashed boxes in the upper panel. The two images on the right side of the middle panel display increased HDAC8 intensity showing no detectable HDAC8 signal associated with nuclei present in ventral roots. On the lower panel, row 3 shows a one-to-one relationship between an HDAC8-positive SC and a STMN2 positive axon (NF-positive), rows 4 and 5 show STMN2-positive small caliber axons (NF-positive) surrounded by HDAC8-positive SC cytoplasm.

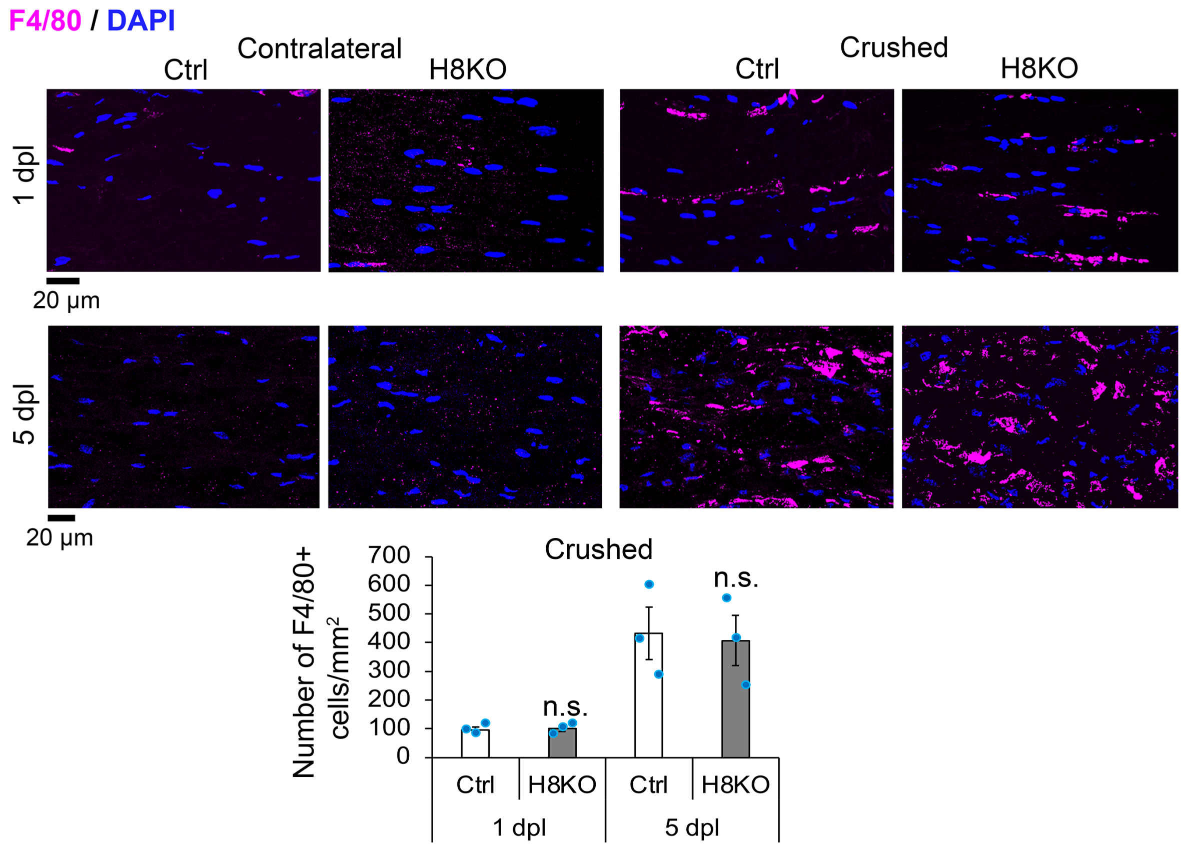

Supplementary Fig. 11. Macrophage numbers after sciatic nerve crush lesion are not affected by the ablation of HDAC8 in SCs. Immunofluorescence of F4/80 (magenta, macrophage marker) and DAPI labeling (blue, nuclei) in crushed and contralateral sciatic nerves of HDAC8 KO (H8KO) and control (Ctrl) mice at 1 and 5 dpl, and quantification of macrophage numbers per mm^2 showing no significant difference between H8KO and Ctrl nerves. Unpaired one-tailed Student's t-tests, n.s.=non-significant, values=mean, error bars=s.e.m., n=3 animals per group, all macrophages present in an entire longitudinal section were quantified per animal.

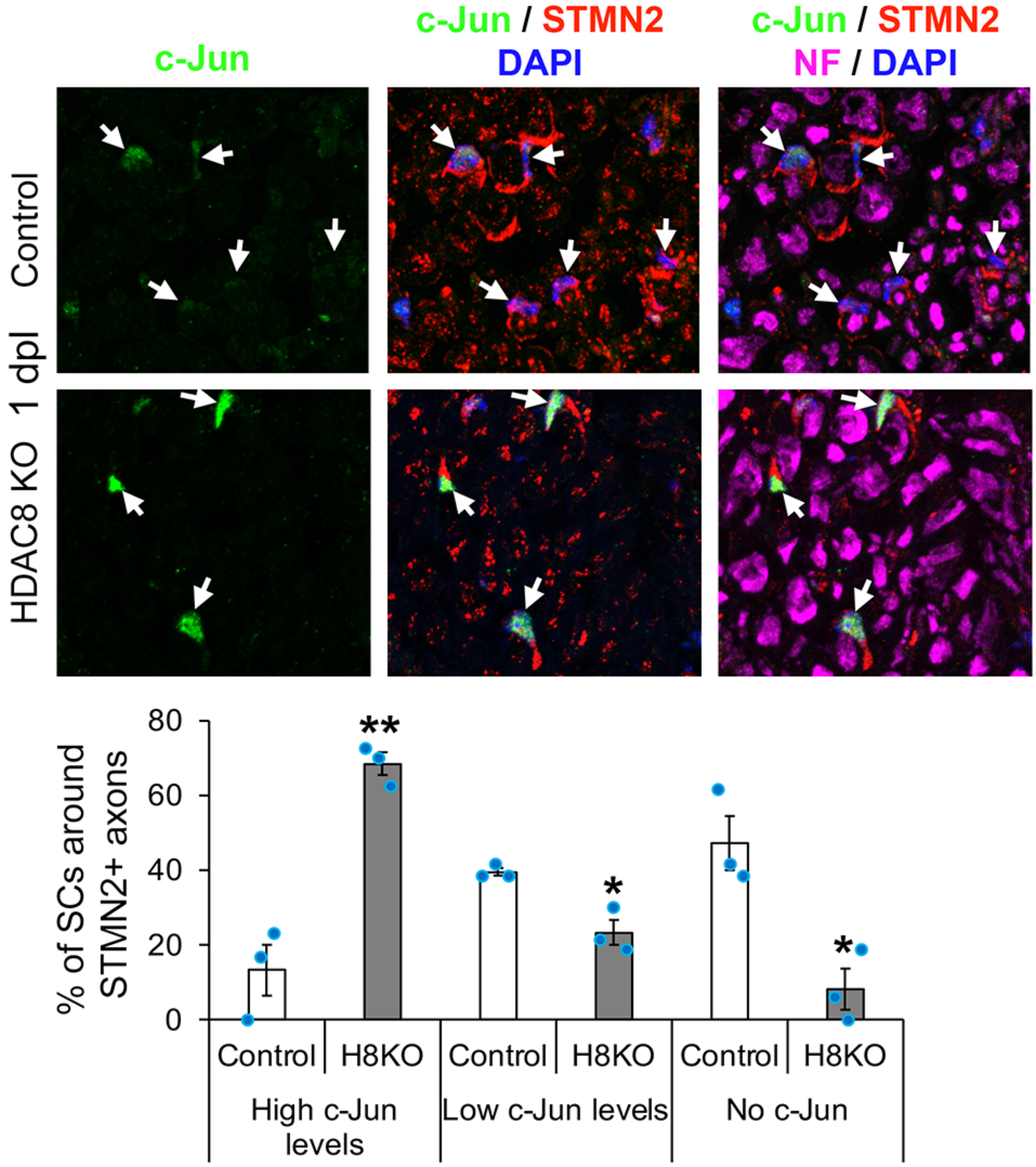

Supplementary Fig. 12. C-Jun is upregulated in SCs surrounding Stathmin-2+ axons. Co-immunofluorescence of c-Jun (green), Stathmin-2 (STMN2, red) and Neurofilament (NF, magenta), and DAPI labeling (blue, nuclei) in crushed sciatic nerves of HDAC8 KO (H8KO) and Control mice at 1 dpl, and quantification of the percentage of SCs surrounding STMN2-positive axons (white arrows) expressing high levels (above a fixed intensity threshold) or low levels of c-Jun or no c-Jun. Unpaired two-tailed Student's t-tests, p values: *<0.05, **<0.01, values=mean, error bars=s.e.m., n=3 animals per group, all SCs surrounding STMN2-positive axons in 3 different cross sections per animal were quantified. Sections were made at the same distance distal to the lesion site.

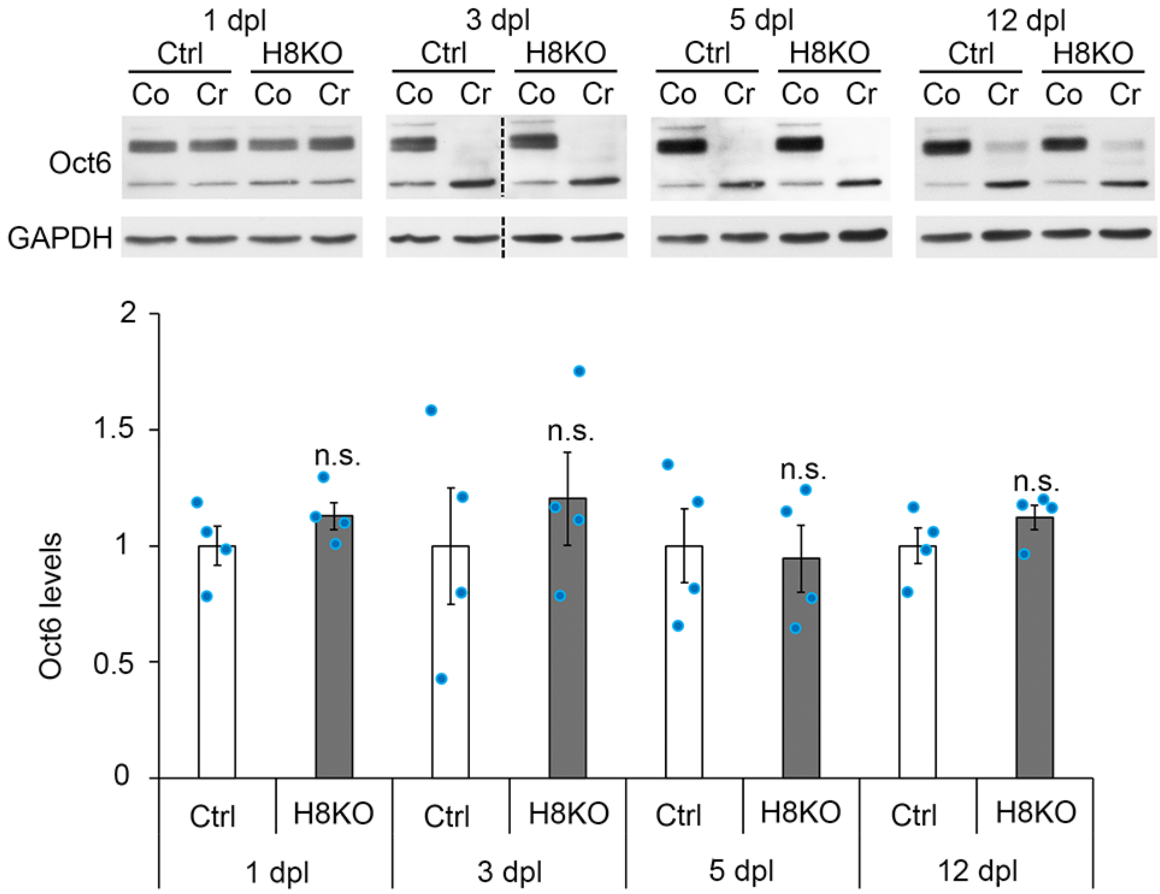

Supplementary Fig. 13. Oct6 levels are not affected by HDAC8 ablation. Oct6 Western blot and quantification normalized to GAPDH at 1, 3, 5 and 12 dpl in lysates of contralateral (Co) and crushed (Cr) mouse sciatic nerves, showing similar Oct6 levels in crushed sciatic nerves of HDAC8 KO (H8KO) and Control (Ctrl) mice. Unpaired one-tailed Student's t-tests, n.s.=non significant, values=mean, error bars=s.e.m., n= 4 animals per group and per time point. Dashed lines indicate that samples were run on the same gel but not on consecutive lanes.

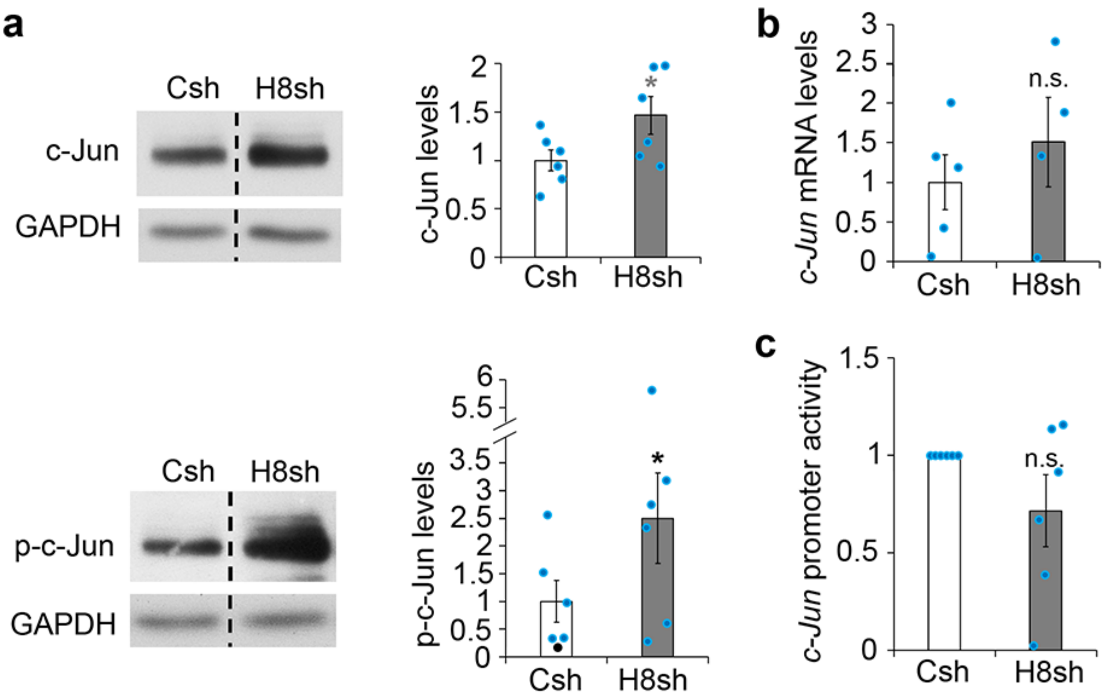

Supplementary Fig. 14. HDAC8-mediated regulation of c-Jun in normoxia. a Western blots of c-Jun and phospho-c-Jun (p-c-Jun) on lysates of rat SCs cultured under conditions mimicking the conversion into the repair phenotype in normoxia and transduced with lentiviruses carrying either a HDAC8-specific shRNA (H8sh) or a control shRNA (Csh), and quantification normalized to GAPDH showing increased c-Jun and p-c-Jun levels in cells where HDAC8 is downregulated. Paired one-tailed (grey asterisk) or two-tailed (black asterisk) Student's t-tests, p value : *<0.05, values=mean, error bars=s.e.m., n= 6 independent experiments per group. Dashed lines indicate that samples were run on the same gel but not on consecutive lanes. **b** Quantification of *c-Jun* mRNA levels by qRT-PCR in primary rat SCs cultured as above (a), showing no significant difference in *c-Jun* expression in cells where HDAC8 was downregulated compared to control. Unpaired one-tailed Student's t-tests, n.s.=non significant, values=mean, error bars=s.e.m., n=4 (H8sh) or 5 (Csh) independent experiments per group. **c** Quantification of *c-Jun* promoter activity by luciferase gene reporter assay in cells cultured as above (a), showing similar activity in cells where HDAC8 was downregulated compared to control cells. Paired one-tailed Student's t-tests, n.s.=non significant, values=mean, error bars=s.e.m., n=6 independent experiments per group.

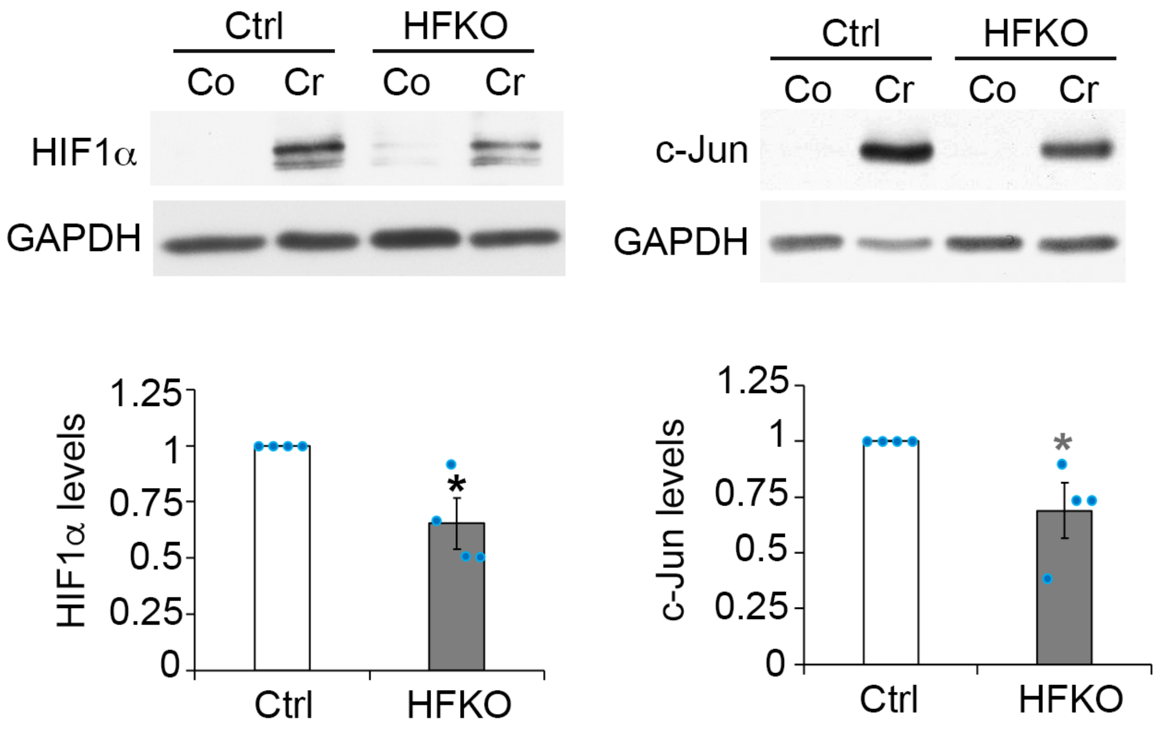

Supplementary Fig. 15. Ablation of HIF1α in SCs leads to decreased c-Jun upregulation after lesion. HIF1α or c-Jun Western blot and quantification normalized to GAPDH at 3 dpl, showing decreased c-Jun levels in crushed sciatic nerves of HIF1α KO mice (HFKO) compared to Control mice (Ctrl). Paired one-tailed (grey asterisk) or two-tailed (black asterisk) Student's t-tests, p value: *<0.05, values=mean, error bars=s.e.m., n=4 animals per group.

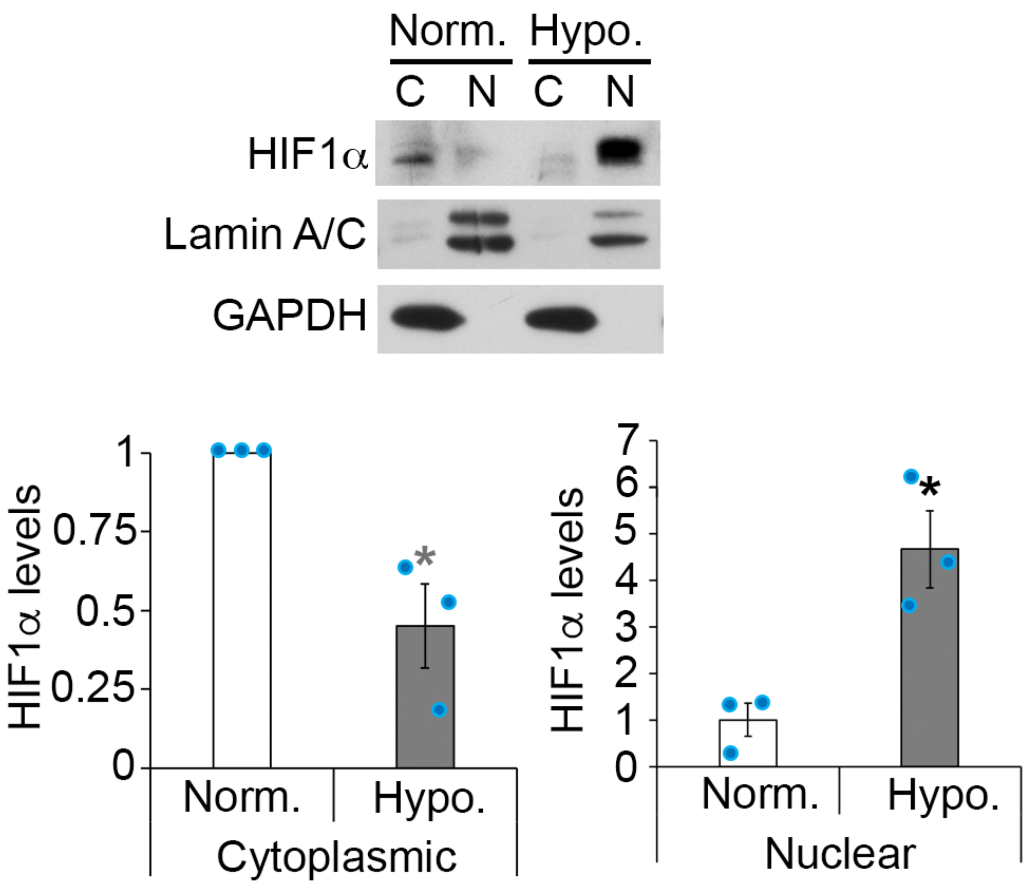

Supplementary Fig. 16. HIF1α upregulation and translocation to nuclear compartment under hypoxia. HIF1α Western blot after subcellular fractionation of cytoplasmic (C) and nuclear (N) fractions of primary rat SCs cultured under normoxia (Norm.) or hypoxia (Hypo., $CoCl_2$ for 16 h) in conditions mimicking the conversion into the repair phenotype. GAPDH and Lamin A/C are used as markers of the cytoplasmic and nuclear fractions, respectively. The graphs represent the quantification of HIF1α levels in normoxia compared to hypoxia in the cytoplasmic and the nuclear fractions, and show a decrease of HIF1α in the cytoplasm and a strong increase in the nucleus under hypoxia. Paired (Cytoplasmic) or unpaired (Nuclear) one-tailed (Cytoplasmic) or two-tailed (Nuclear) Student's t-tests, p values : $*<0.05$, values=mean, error bars=s.e.m., n=3 independent experiments per group.

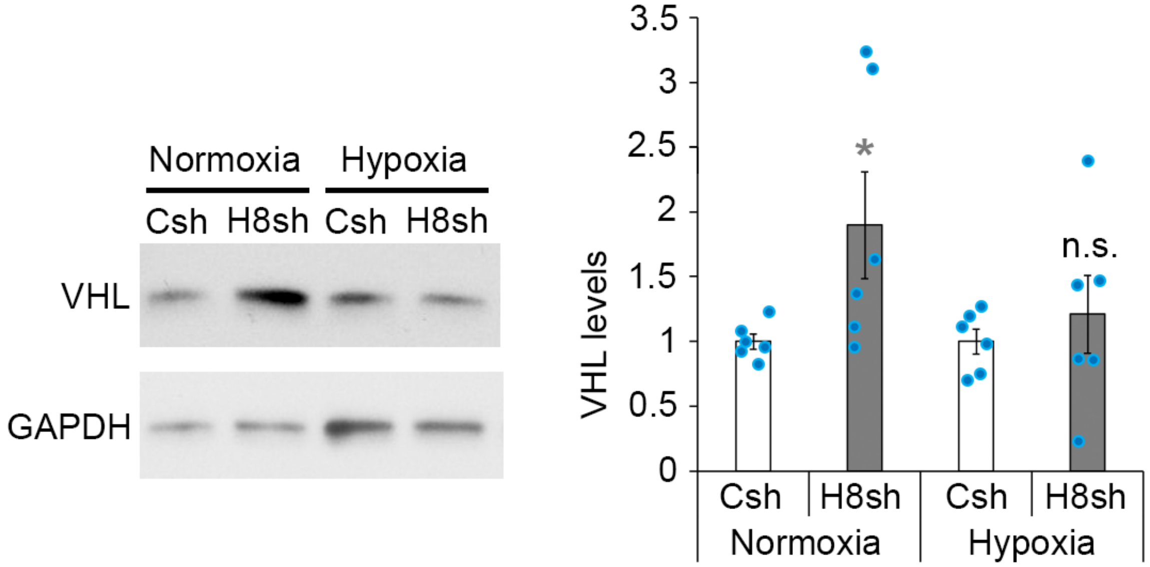

Supplementary Fig. 17. HDAC8 knockdown does not induce VHL downregulation. Western blot of VHL on lysates of rat SCs cultured under conditions mimicking the conversion into the repair phenotype in normoxia or hypoxia ($CoCl_2$, 16 h) and transduced with lentiviruses carrying either a HDAC8-specific shRNA (H8sh) or a control shRNA (Csh), and quantification normalized to GAPDH showing increased VHL levels in normoxia and no difference in hypoxia in cells where HDAC8 is downregulated compared to control cells. Paired one-tailed Student's t-tests, p value : *<0.05, n.s.=non significant, values=mean, error bars=s.e.m., n=6 independent experiments per group.

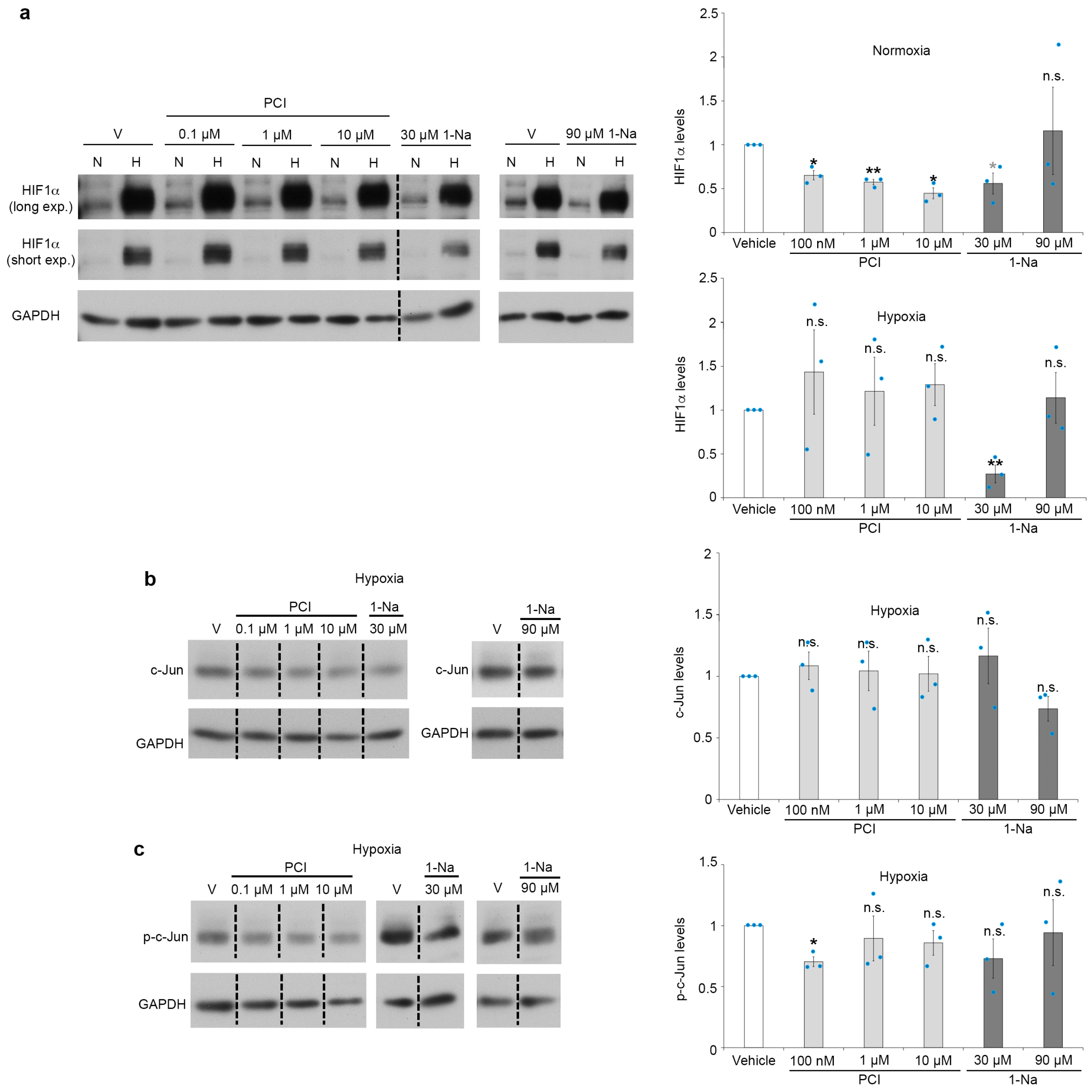

Supplementary Fig. 18. HDAC8 inhibitors do not increase the levels of HIF1α, c-Jun or phospho-c-Jun. Western blots of HIF1α (**a**), c-Jun (**b**) and phospho-c-Jun (p-c-Jun, **c**) in lysates of primary rat SCs cultured under normoxia (N) or hypoxia (H) in conditions mimicking the conversion into the repair phenotype, and incubated for 24 h with two different HDAC8 inhibitors, PCI-34051 and 1-Naphthohydroxamic acid, at different concentrations, or with the vehicle (V). The graphs show the quantification of HIF1α (a), c-Jun (b) and p-c-Jun (c) levels normalized to GAPDH. Dashed lines indicate that samples were run on the same gel but not on consecutive lanes. Paired one-tailed (grey asterisks) or two-tailed (black asterisk) Student's t-tests, p values: *<0.05, **<0.01, n.s.=non significant, values=mean, error bars=s.e.m., n=3 independent experiments per group.

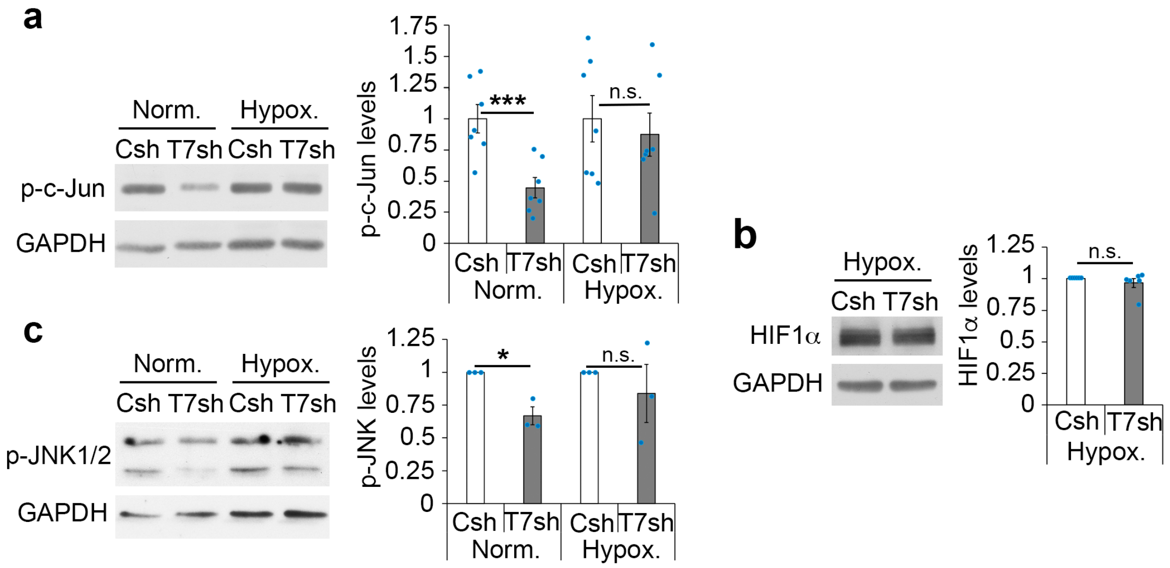

Supplementary Fig. 19. TRAF7 knockdown affects JNK and c-Jun phosphorylation in normoxia. Western blots of phospho-c-Jun (p-c-Jun, **a**), HIF1α (**b**) and phospho-JNK (p-JNK, **c**) in primary rat SCs cultured under normoxia (Norm.) or hypoxia (Hypox.) in conditions mimicking the conversion into the repair phenotype. The graphs show that TRAF7 knockdown leads to decreased levels of p-c-Jun and p-JNK (normalized to GAPDH) in normoxia but does not affect protein levels in hypoxia. Paired two-tailed (black asterisks) or one-tailed Student's t-tests, p values: *<0.05, ***<0.001, n.s.=non significant, values=mean, error bars=s.e.m., n=7 (a), 6 (b) or 3 (c) independent experiments per group.

Fig. S1a

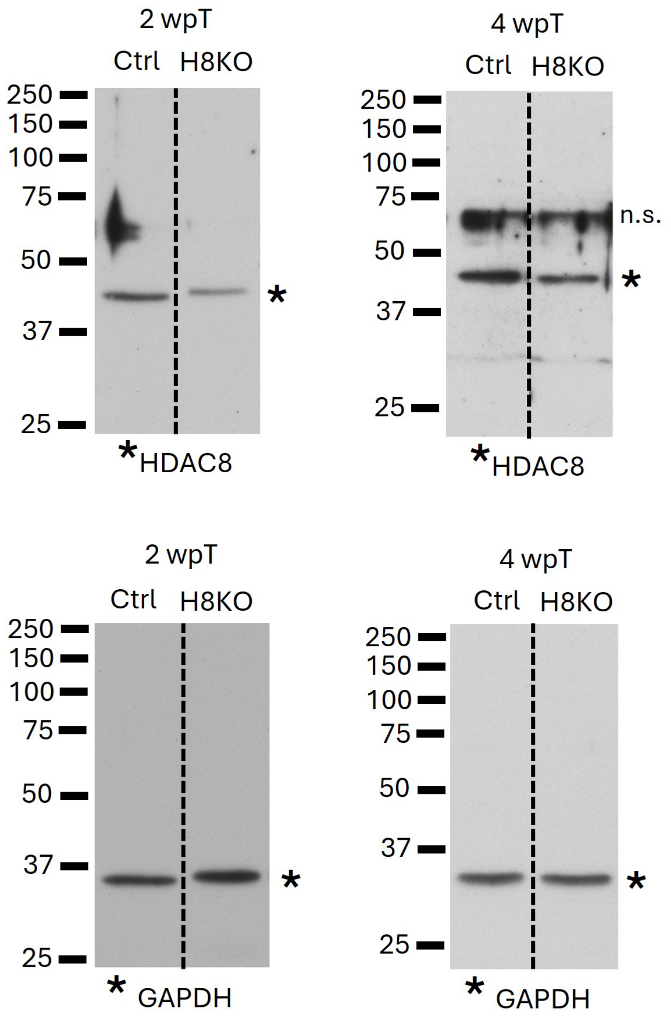

Fig S1b

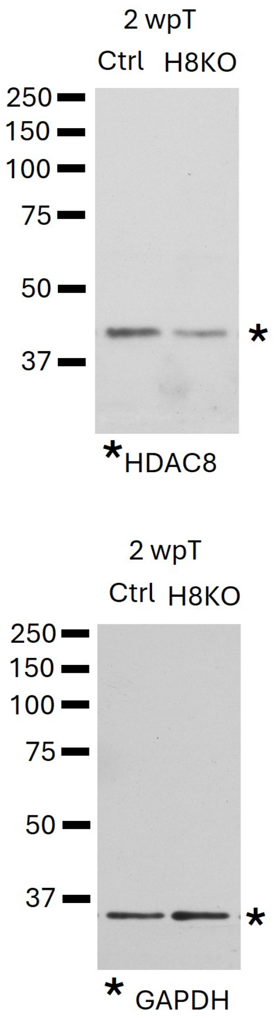

Full blots Supplementary Fig. 1

Fig. S5b

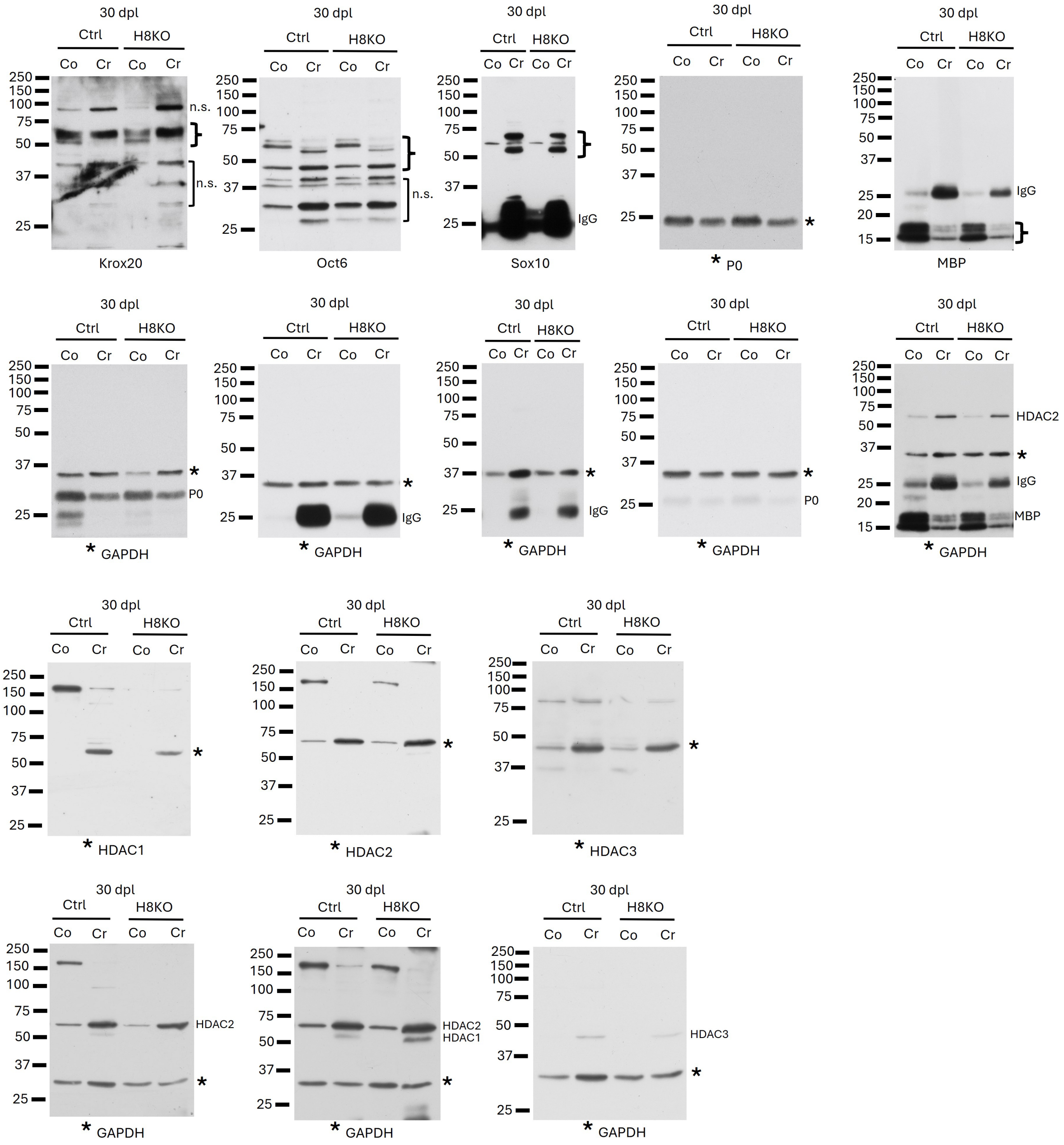

Full blots Supplementary Fig. 5

Fig. S8a

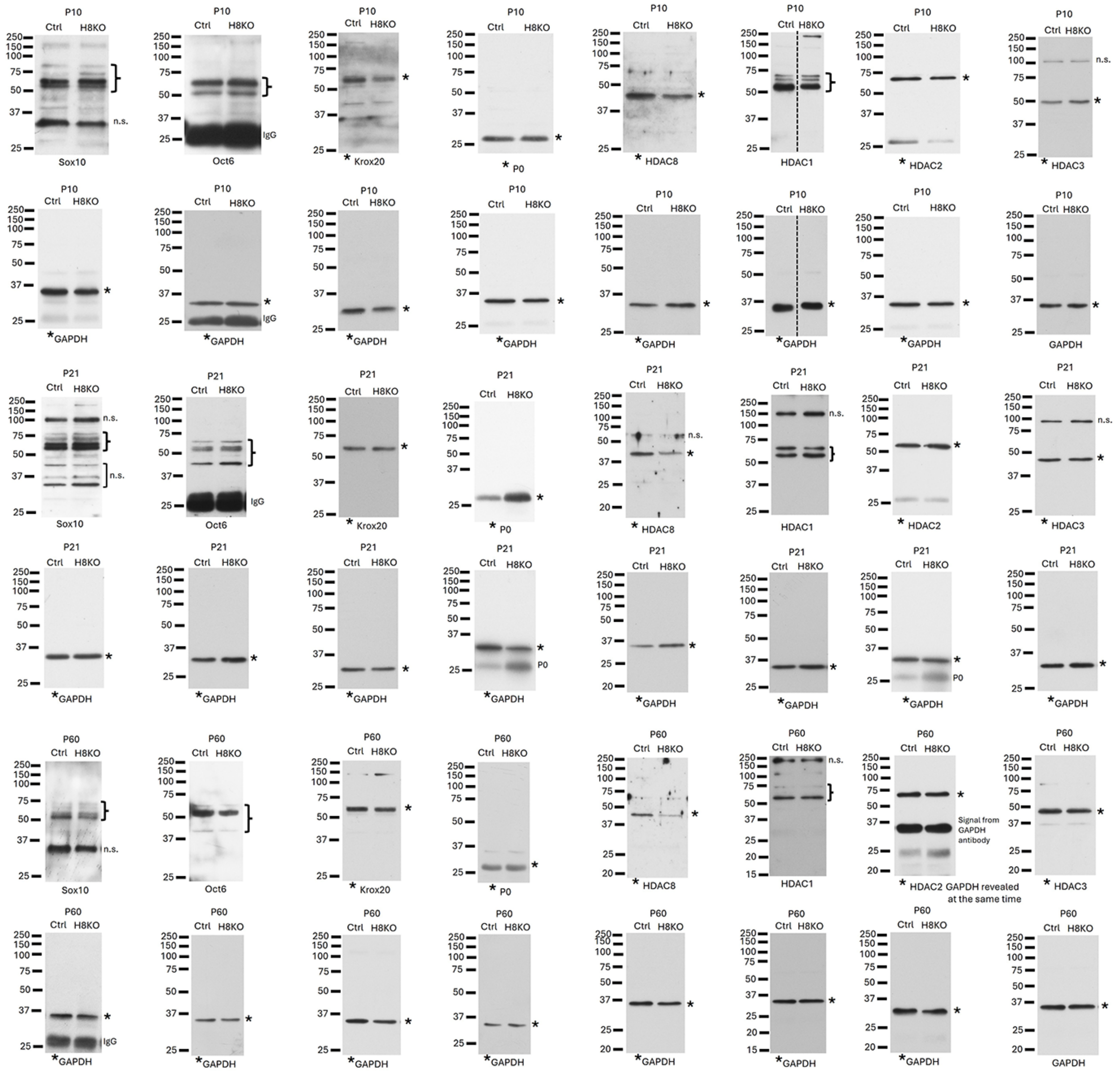

Full blots Supplementary Fig. 8

Fig. S13

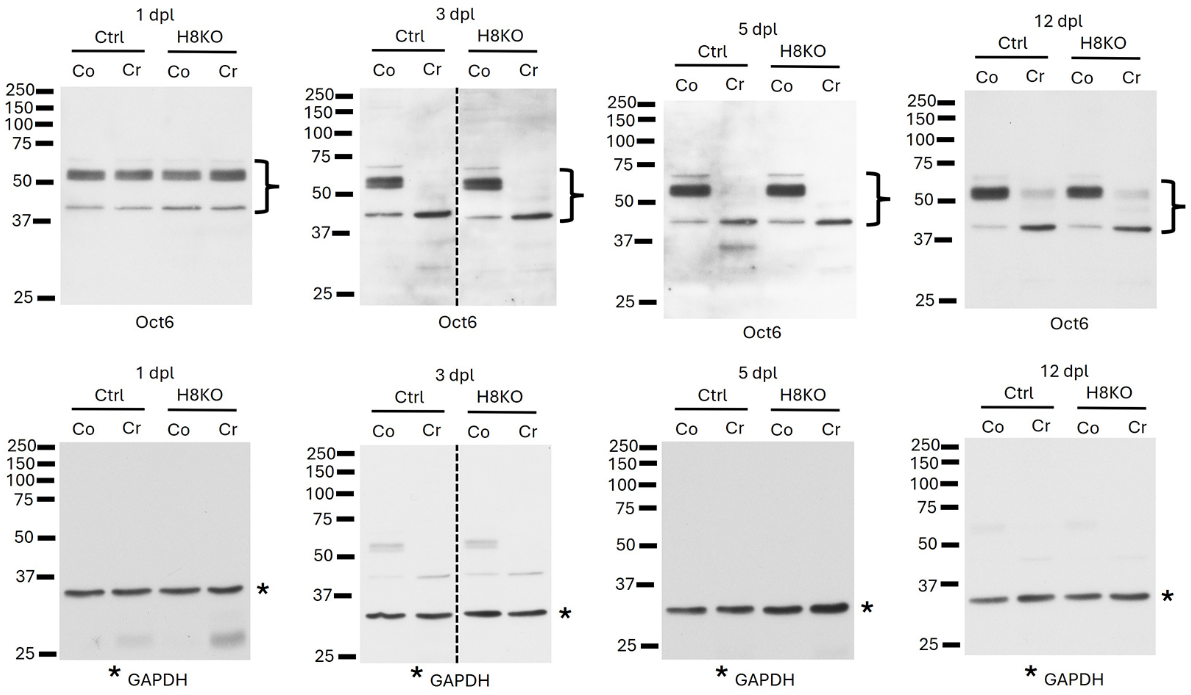

Full blots Supplementary Fig. 13

Fig. S14 a

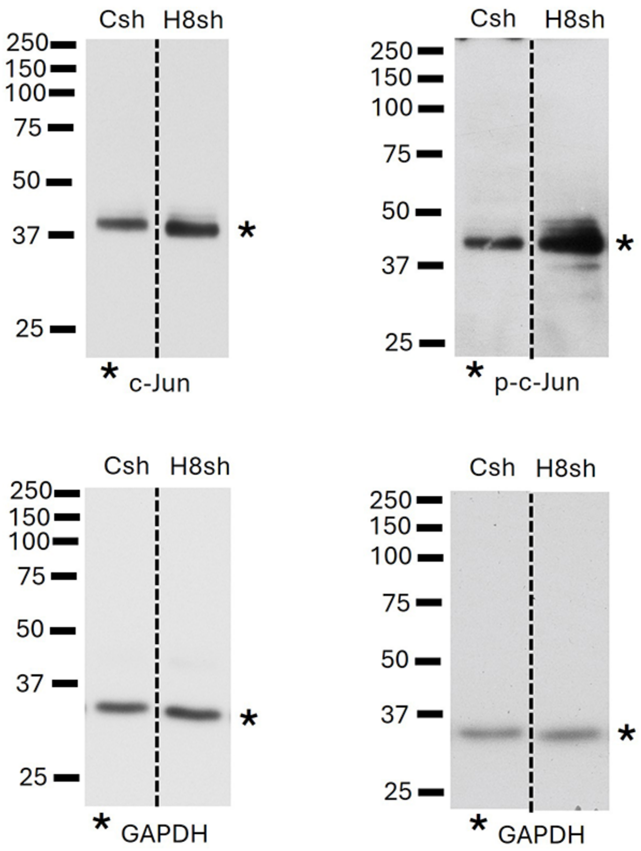

Full blots Supplementary Fig. 14

Fig. S15

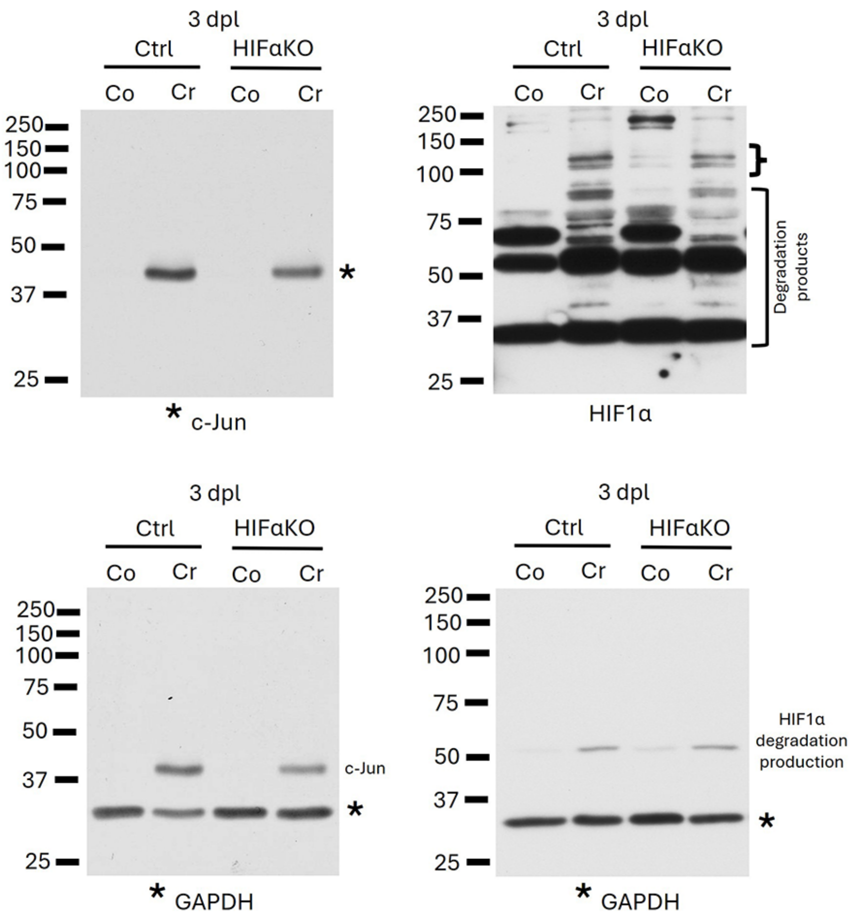

Full blots Supplementary Fig. 15

Fig. S16

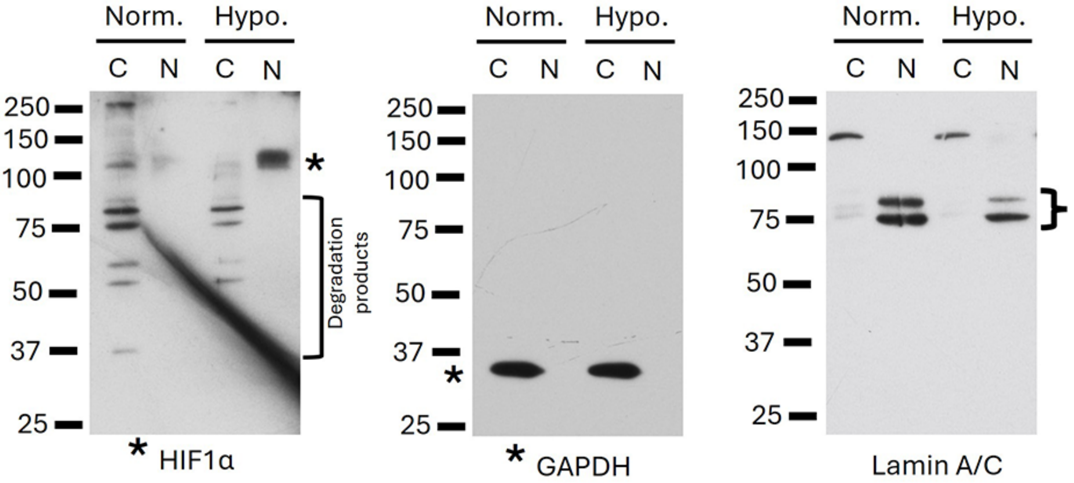

Full blots Supplementary Fig. 16

Fig. S17

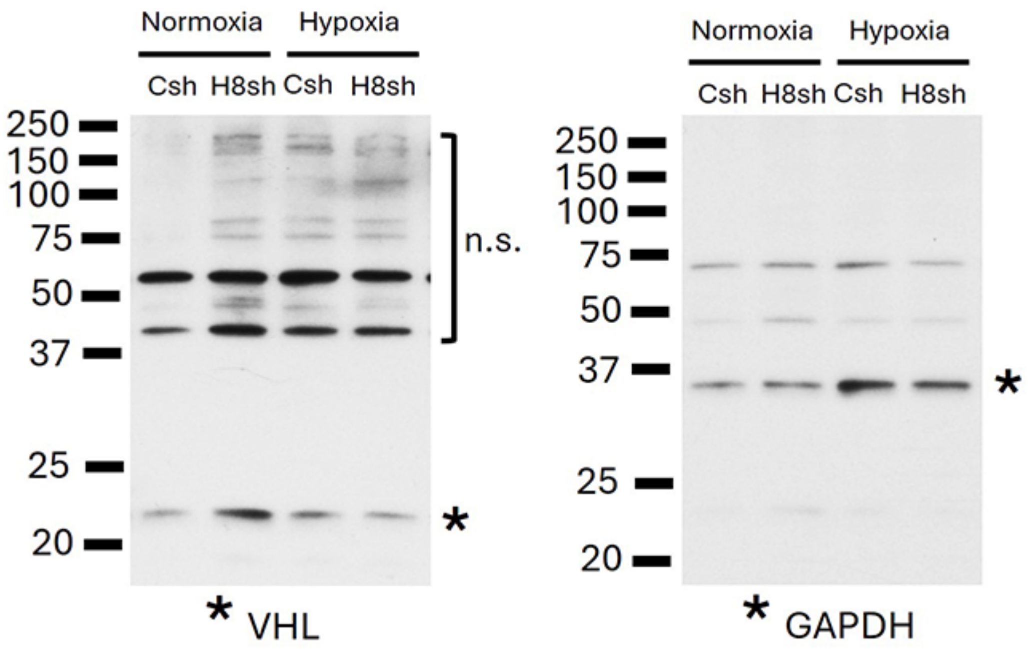

Full blots Supplementary Fig. 17

Fig. S18a

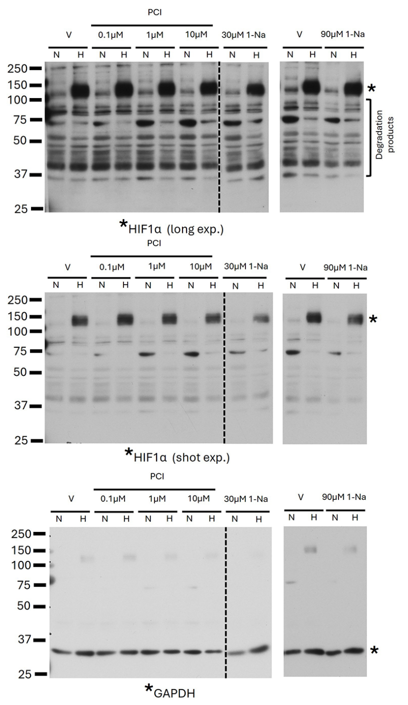

Fig. S18b

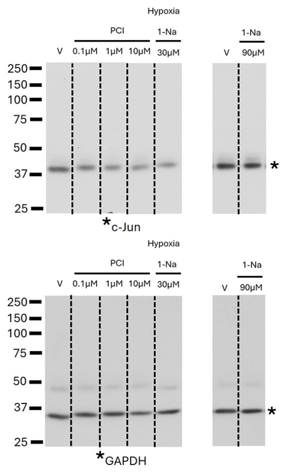

Fig. S18c

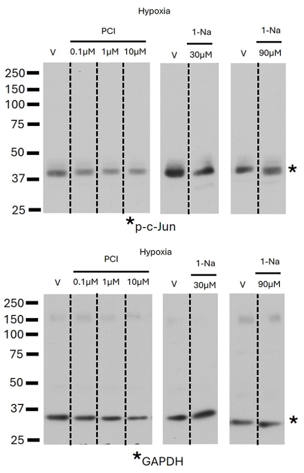

Full blots Supplementary Fig. 18

Fig. S19a

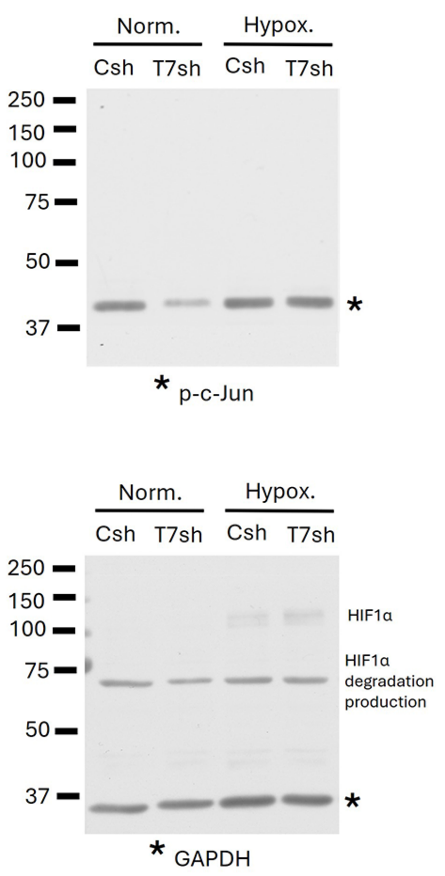

Fig. S19b

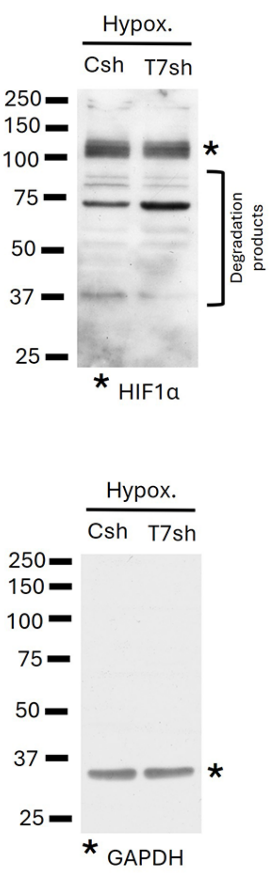

Fig. S19c

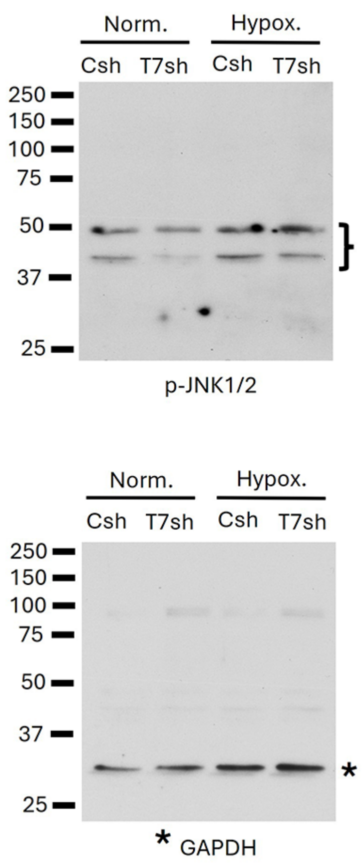

Full blots Supplementary Fig. 19
