# Supplementary material for: Hypoxia-induced conversion of sensory Schwann cells into repair cells is regulated by HDAC8
Source: Nat Commun. 2025 Jan 9;16:515. doi: 10.1038/s41467-025-55835-9 (PMC11711395; doi:10.1038/s41467-025-55835-9)
Supplement: Supplementary file 2 — Description of Additional Supplementary Files [file 41467_2025_55835_MOESM2_ESM.pdf]

Description of Additional Supplementary Files

Supplementary Data 1. HDAC8 putative binding partners. Mass spectrometry analysis of HDAC8 putative binding partners in contralateral (CO) and crushed (CR) mouse sciatic nerves at 1 dpl after immunoprecipitation with HDAC8-specific antibody or a control IgG, sequence of TRAF7 and HDAC8 identified peptides, and GO analysis of enriched terms.
