# Supplementary material for: Hypoxia-induced conversion of sensory Schwann cells into repair cells is regulated by HDAC8
Source: Nat Commun. 2025 Jan 9;16:515. doi: 10.1038/s41467-025-55835-9 (PMC11711395; doi:10.1038/s41467-025-55835-9)
Supplement: Supplementary file 4 — Reporting Summary [file 41467_2025_55835_MOESM4_ESM.pdf]

nature portfolio

Corresponding author(s): Claire Jacob

Last updated by author(s): Dec 2, 2024

Reporting Summary

Nature Portfolio wishes to improve the reproducibility of the work that we publish. This form provides structure for consistency and transparency in reporting. For further information on Nature Portfolio policies, see our Editorial Policies and the Editorial Policy Checklist.

Statistics

For all statistical analyses, confirm that the following items are present in the figure legend, table legend, main text, or Methods section.

n/a	Confirmed	
☐	☒	The exact sample size (*n*) for each experimental group/condition, given as a discrete number and unit of measurement
☐	☒	A statement on whether measurements were taken from distinct samples or whether the same sample was measured repeatedly
☐	☒	The statistical test(s) used AND whether they are one- or two-sided<br>*Only common tests should be described solely by name; describe more complex techniques in the Methods section.*
☒	☐	A description of all covariates tested
☒	☐	A description of any assumptions or corrections, such as tests of normality and adjustment for multiple comparisons
☐	☒	A full description of the statistical parameters including central tendency (e.g. means) or other basic estimates (e.g. regression coefficient) AND variation (e.g. standard deviation) or associated estimates of uncertainty (e.g. confidence intervals)
☐	☒	For null hypothesis testing, the test statistic (e.g. *F*, *t*, *r*) with confidence intervals, effect sizes, degrees of freedom and *P* value noted<br>*Give P values as exact values whenever suitable.*
☒	☐	For Bayesian analysis, information on the choice of priors and Markov chain Monte Carlo settings
☒	☐	For hierarchical and complex designs, identification of the appropriate level for tests and full reporting of outcomes
☒	☐	Estimates of effect sizes (e.g. Cohen's *d*, Pearson's *r*), indicating how they were calculated

Our web collection on statistics for biologists contains articles on many of the points above.

Software and code

Policy information about availability of computer code

Data collection	VisiView® 6.0
Data analysis	MaxQuant using Perseus software version 1.6.2.10, Fiji version 1.0, Microsoft Excel for Mac version 16.91, Adobe Photoshop CC 25.6.0 Release

For manuscripts utilizing custom algorithms or software that are central to the research but not yet described in published literature, software must be made available to editors and reviewers. We strongly encourage code deposition in a community repository (e.g. GitHub). See the Nature Portfolio guidelines for submitting code & software for further information.

Data

Policy information about availability of data

All manuscripts must include a data availability statement. This statement should provide the following information, where applicable:

- Accession codes, unique identifiers, or web links for publicly available datasets
- A description of any restrictions on data availability
- For clinical datasets or third party data, please ensure that the statement adheres to our policy

The mass spectrometry proteomics data have been deposited to the ProteomeXchange Consortium via the PRIDE [1] partner repository with the dataset identifier PXD046582. Source data including uncropped blots of main Figures and raw data of all figures are provided with this paper. Uncropped blots of Supplementary Figures are presented at the end of the Supplementary Information file.

Research involving human participants, their data, or biological material

Policy information about studies with human participants or human data. See also policy information about sex, gender (identity/presentation), and sexual orientation and race, ethnicity and racism.

Reporting on sex and gender	n/a
Reporting on race, ethnicity, or other socially relevant groupings	n/a
Population characteristics	n/a
Recruitment	n/a
Ethics oversight	n/a

Note that full information on the approval of the study protocol must also be provided in the manuscript.

Field-specific reporting

Please select the one below that is the best fit for your research. If you are not sure, read the appropriate sections before making your selection.

☒ Life sciences ☐ Behavioural & social sciences ☐ Ecological, evolutionary & environmental sciences

For a reference copy of the document with all sections, see nature.com/documents/nr-reporting-summary-flat.pdf

Life sciences study design

All studies must disclose on these points even when the disclosure is negative.

Sample size	Sample size was determined by the minimal number of animals or individual experiment required to obtain statistically significant results and increased in some cases to improve confidence in the results obtained, n=3 to 12. Sample size was calculated for a power of 0.8.
Data exclusions	No animal or data point was excluded from the analysis.
Replication	All data presented were successfully reproduced.
Randomization	To minimize heterogeneity between groups, we used littermate animals (same sex whenever possible) as controls of KO animals.
Blinding	The experimenter collecting data (e.g. behavioral analyses) or analyzing data (e.g. Immunofluorescence) was blinded to the genotype and received only the animal number given at birth by the animal caretaker. Analysis of data were done at least twice and by different persons independently.

Reporting for specific materials, systems and methods

We require information from authors about some types of materials, experimental systems and methods used in many studies. Here, indicate whether each material, system or method listed is relevant to your study. If you are not sure if a list item applies to your research, read the appropriate section before selecting a response.

Materials & experimental systems

n/a	Involved in the study	
☐	☒	Antibodies
☐	☒	Eukaryotic cell lines
☒	☐	Palaeontology and archaeology
☐	☒	Animals and other organisms
☒	☐	Clinical data
☒	☐	Dual use research of concern
☒	☐	Plants

Methods

n/a	Involved in the study	
☒	☐	ChIP-seq
☒	☐	Flow cytometry
☒	☐	MRI-based neuroimaging

Antibodies

Antibodies used	All antibodies are described in the Methods section, along with the name of the manufacturer, the catalog number, the

Antibodies used	concentration used and the last lot number used.
Validation	Antibodies used in this study were validated by the manufacturer (validation method shown on the manufacturer's website) and for most of them were additionally validated by references listed on the manufacturer's website. Two antibodies were provided by other labs and were validated in multiple publications and by the lab who produced the antibody.

Eukaryotic cell lines

Policy information about cell lines and Sex and Gender in Research

Cell line source(s)	We used primary rat Schwann cells that we collected and purified ourselves.
Authentication	Identity and purity of our primary Schwann cells were checked for each primary preparation by immunofluorescence of Schwann cell-specific markers (p75, Sox10, Oct6, Krox20, P0, MAG).
Mycoplasma contamination	Mycoplasma contamination was not tested, because of the low incidence of mycoplasma contamination in primary cells, and because mycoplasma contamination results in inefficiency of transfection, which we did not observe in our primary cells.
Commonly misidentified lines (See ICLAC register)	no commonly misidentified cell lines were used in the study.

Animals and other research organisms

Policy information about studies involving animals; ARRIVE guidelines recommended for reporting animal research, and Sex and Gender in Research

Laboratory animals	Male and female mice from mixed genetic background were used. Young adults of 3-4-month old were used.
Wild animals	The study did not involve wild animals.
Reporting on sex	Male and female mice were used. Whenever possible, littermate mice of the same sex were used as controls. We did not identify any sex specific effect, therefore the results presented were collected from both male and female mice.
Field-collected samples	The study did not involve samples collected from the field.
Ethics oversight	Veterinary office of the Canton of Fribourg, Switzerland and Veterinary office (Landesuntersuchungsamt) of Rheinland-Pfalz, Germany, Veterinary office (Landesdirektion) in Sachsen, Germany

Note that full information on the approval of the study protocol must also be provided in the manuscript.

Plants

Seed stocks	n/a
Novel plant genotypes	n/a
Authentication	n/a
