# Supplementary figures and images for: Hypoxia-induced conversion of sensory Schwann cells into repair cells is regulated by HDAC8
Source: Nat Commun. 2025 Jan 9;16:515. doi: 10.1038/s41467-025-55835-9 (PMC11711395; doi:10.1038/s41467-025-55835-9)

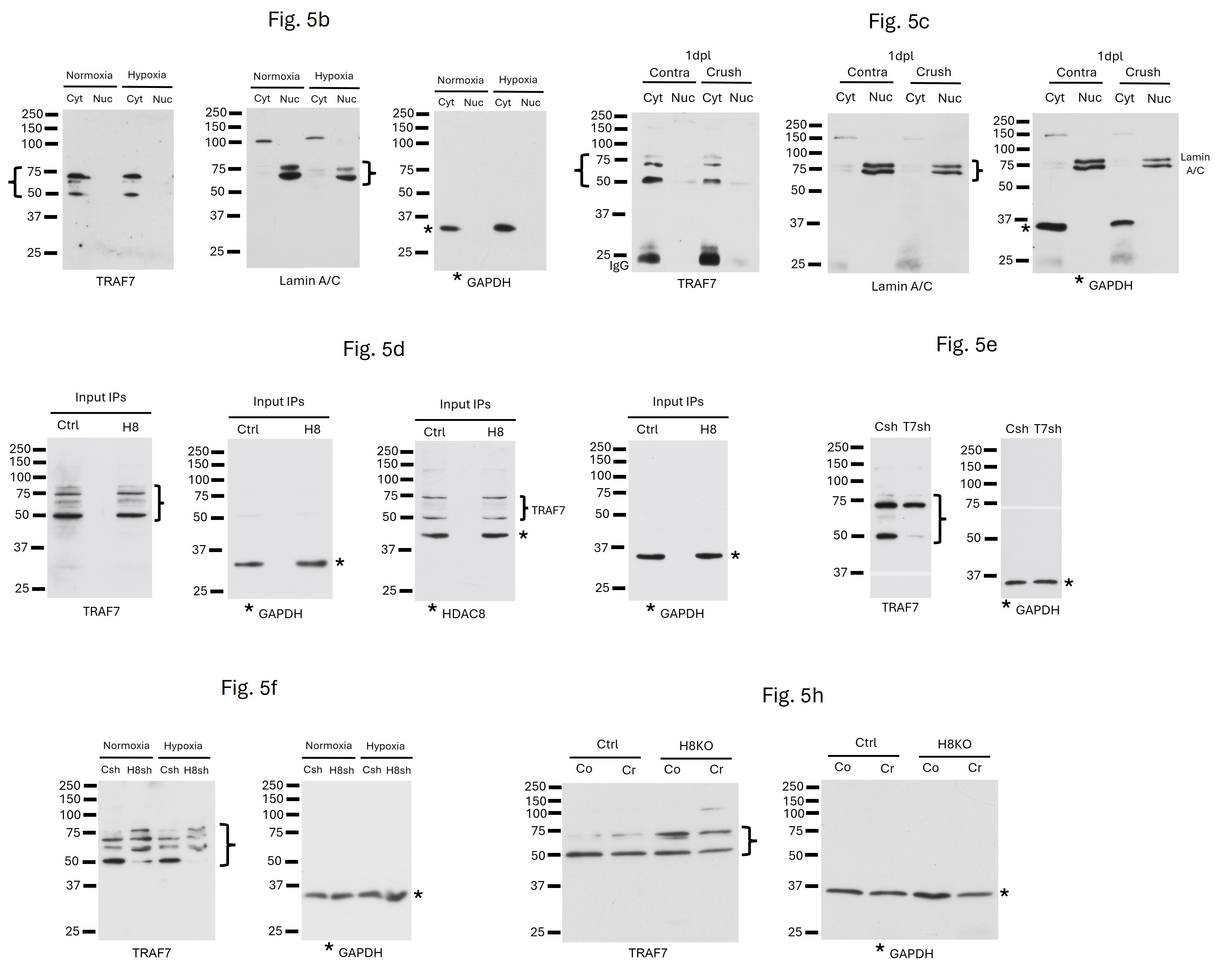

Supplement: Supplementary file 6 — Source data [file 41467_2025_55835_MOESM6_ESM.zip › Source Data files/Fig. 5_full blots.tif]

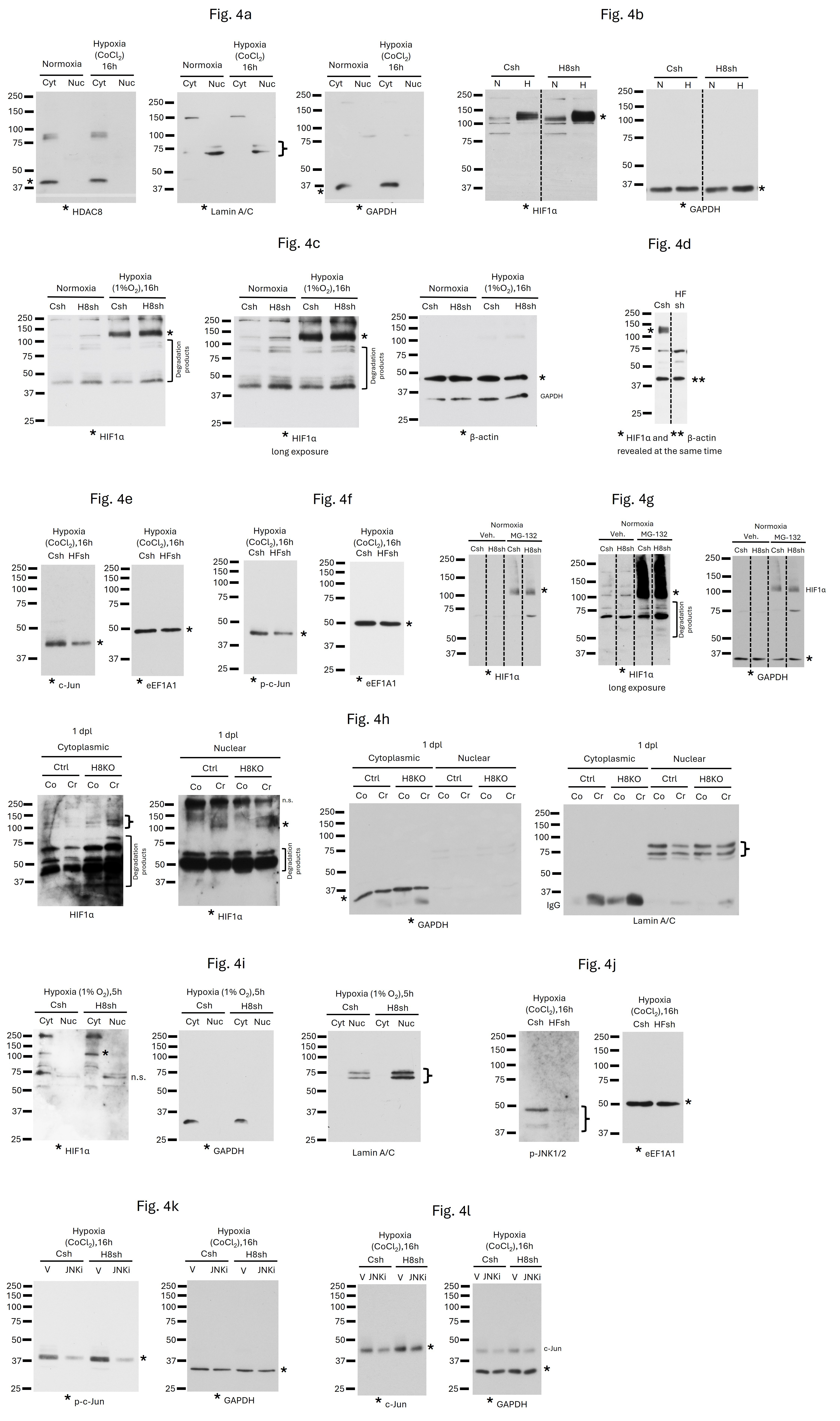

Supplement: Supplementary file 6 — Source data [file 41467_2025_55835_MOESM6_ESM.zip › Source Data files/Fig. 4 full blots.jpg]

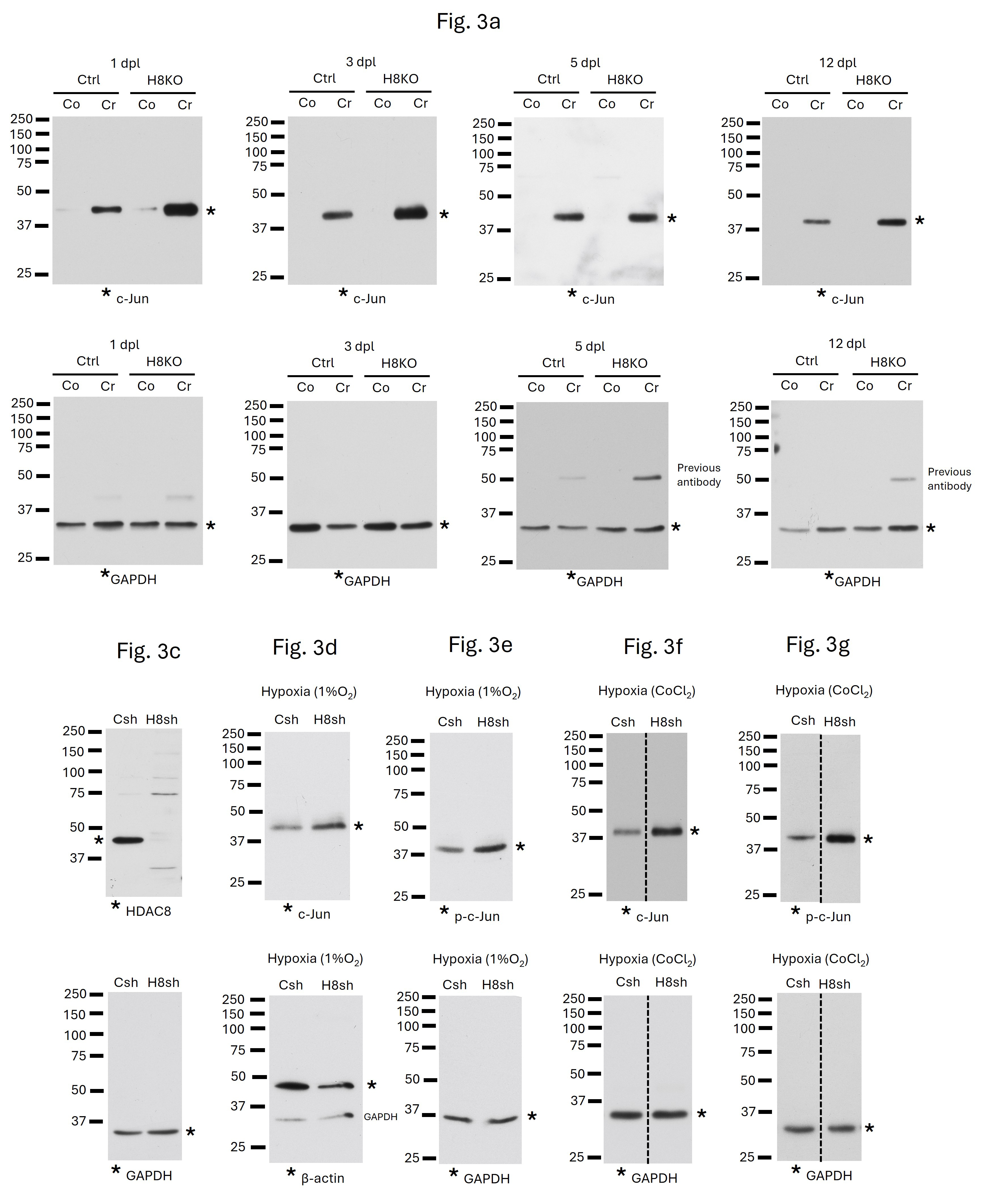

Supplement: Supplementary file 6 — Source data [file 41467_2025_55835_MOESM6_ESM.zip › Source Data files/Fig. 3 full blots.jpg]

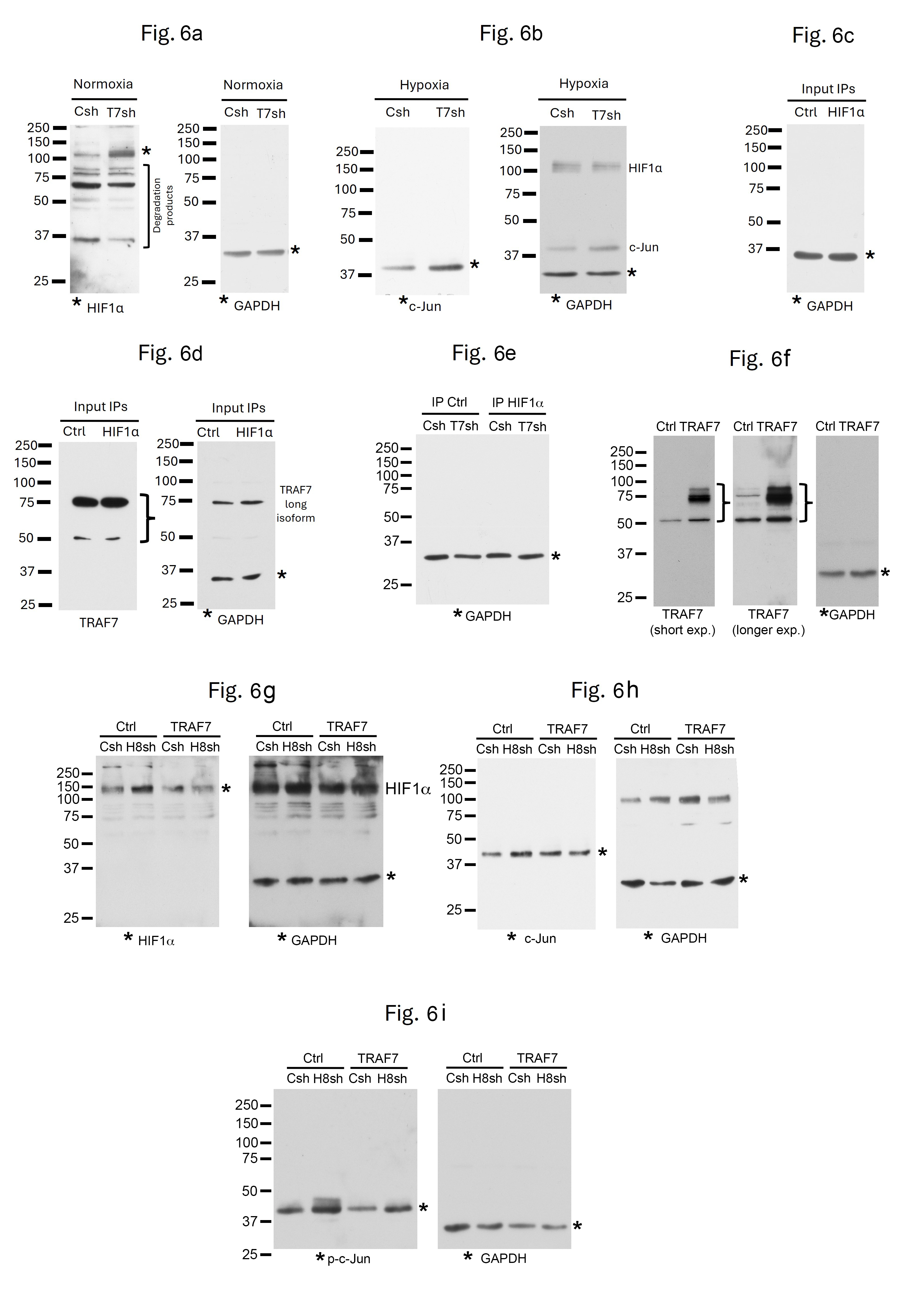

Supplement: Supplementary file 6 — Source data [file 41467_2025_55835_MOESM6_ESM.zip › Source Data files/Fig. 6 full blots.tif]

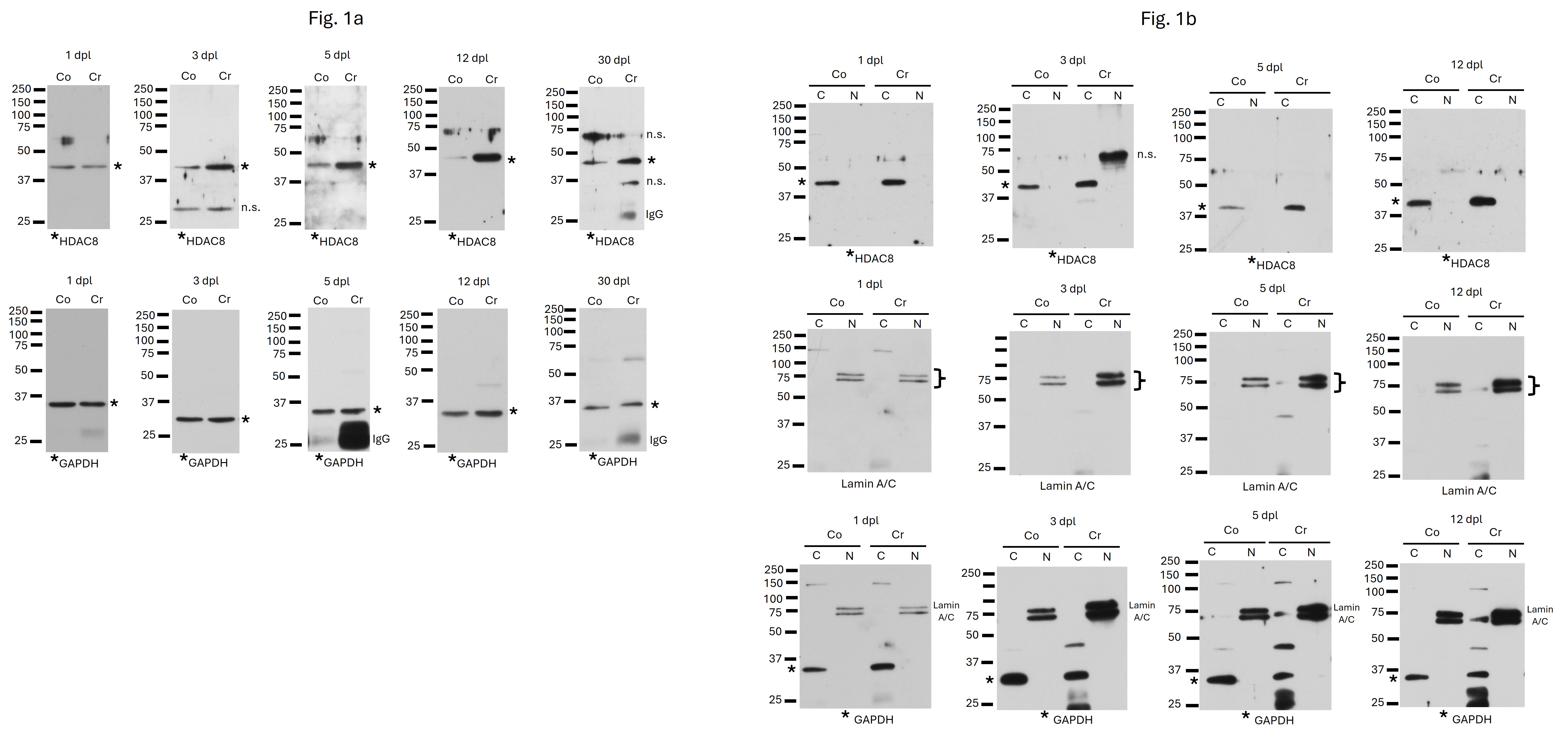

Supplement: Supplementary file 6 — Source data [file 41467_2025_55835_MOESM6_ESM.zip › Source Data files/Fig. 1 full blots.jpg]
